# A T Cell‐Engaging Tumor Organoid Platform for Pancreatic Cancer Immunotherapy

**DOI:** 10.1002/advs.202300548

**Published:** 2023-06-04

**Authors:** Zhuolong Zhou, Kevin Van der Jeught, Yujing Li, Samantha Sharma, Tao Yu, Ishara Moulana, Sheng Liu, Jun Wan, Paul R. Territo, Mateusz Opyrchal, Xinna Zhang, Guohui Wan, Xiongbin Lu

**Affiliations:** ^1^ Department of Medical and Molecular Genetics Indiana University School of Medicine Indianapolis IN 46202 USA; ^2^ Department of Medical and Molecular Genetics Center for Computational Biology and Bioinformatics Indiana University School of Medicine Indianapolis IN 46202 USA; ^3^ Department of Radiology and Imaging Sciences Indiana University School of Medicine Indianapolis IN 46202 USA; ^4^ Division of Hematology/Oncology Department of Medicine Melvin and Bren Simon Comprehensive Cancer Center Indiana University School of Medicine Indianapolis IN 46202 USA; ^5^ Department of Medical and Molecular Genetics Melvin and Bren Simon Comprehensive Cancer Center Indiana University School of Medicine Indianapolis IN 46202 USA; ^6^ School of Pharmaceutical Sciences Sun Yat‐Sen University Guangzhou 510006 China; ^7^ Department of Medical and Molecular Genetics Center for Computational Biology and Bioinformatics Melvin and Bren Simon Comprehensive Cancer Center Indiana University School of Medicine Indianapolis IN 46202 USA

**Keywords:** epigenetic inhibitors, high‐throughput drug screen, immunotherapy, pancreatic ductal adenocarcinoma, patient‐derived organoid, tumor antigen presentation, tumor organoid

## Abstract

Pancreatic ductal adenocarcinoma (PDA) is a clinically challenging disease with limited treatment options. Despite a small percentage of cases with defective mismatch DNA repair (dMMR), PDA is included in the most immune‐resistant cancer types that are poorly responsive to immune checkpoint blockade (ICB) therapy. To facilitate drug discovery combating this immunosuppressive tumor type, a high‐throughput drug screen platform is established with the newly developed T cell‐incorporated pancreatic tumor organoid model. Tumor‐specific T cells are included in the pancreatic tumor organoids by two‐step cell packaging, fully recapitulating immune infiltration in the immunosuppressive tumor microenvironment (TME). The organoids are generated with key components in the original tumor, including epithelial, vascular endothelial, fibroblast and macrophage cells, and then packaged with T cells into their outside layer mimicking a physical barrier and enabling T cell infiltration and cytotoxicity studies. In the PDA organoid‐based screen, epigenetic inhibitors ITF2357 and I‐BET151 are identified, which in combination with anti‐PD‐1 based therapy show considerably greater anti‐tumor effect. The combinatorial treatment turns the TME from immunosuppressive to immunoactive, up‐regulates the MHC‐I antigen processing and presentation, and enhances the effector T cell activity. The standardized PDA organoid model has shown great promise to accelerate drug discovery for the immunosuppressive cancer.

## Graphical abstract

Pancreatic ductal adenocarcinoma is one of the most immune‐resistant cancer types. To combat this immunosuppressive tumor type, a new T cell‐incorporated pancreatic tumor organoid model is established, fully recapitulating immune infiltration in the tumor microenvironment. In a drug screen, epigenetic inhibitors, ITF2357, and I‐BET151 are identified, which significantly promote the efficacy of anti‐PD‐1 based immune checkpoint blockade therapy.

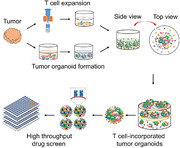

## 1 Introduction

Pancreatic ductal adenocarcinoma (PDA) is an extremely devastating malignancy accounting for ≈90% of all pancreatic cancers.^[^ ^1^ ^]^ In the past two decades, there has been low and minimal change in survival of patients with PDA from surgery, chemotherapy and radiotherapy as many patients have pancreatic tumors unresectable or metastasized at diagnosis.^[^ ^2^ ^]^ PDA processes a desmoplastic and immunosuppressive tumor microenvironment (TME) composed by a dense stroma composition with abundant immunosuppressive cells (myeloid‐derived suppressive cells and regulatory T cells), cancer‐associated fibroblasts and endothelial cells.^[^ ^3^ ^]^ The initiation of PDA with such unique immunosuppressive TME is commonly believed to be the genetic changes and accumulation of oncogenic mutations (such as mutant KRAS and p53).^[^ ^4^, ^5^ ^]^ Immunotherapy, particularly the PD‐1/PD‐L1 antibody‐mediated immune checkpoint blockade (ICB) therapy, has been proven in recent years as a promising treatment strategy for many cancers along with better safety advantage.^[^ ^6^ ^]^ However, except for a small portion of PDA cases (≈1%) with defective mismatch DNA repair (dMMR), immune monotherapies for microsatellite‐stable PDA have been ineffective in clinic.^[^ ^7^ ^]^ Immunotherapy combined with other therapy modalities is likely to unleash the antitumor capacity of the immune system against this hardest target in cancer care.^[^ ^8^ ^]^ The result of a recent clinical trial showed that patients with advanced pancreatic cancer with a combinational treatment (PD‐1 antibody nivolumab plus two chemotherapy drugs, nab‐paclitaxel, and gemcitabine) had a one‐year survival rate of 57.7%, significantly greater than the historical average survival of 35%.^[^ ^8^ ^]^ The revelation of PDA genomic landscapes and immune profiles have accelerated the development of targeted immunotherapy.^[^ ^2^ ^]^ Limited response of PDA to ICB‐based therapy can be ascribed to i) suppression of immune recognition on tumor antigens; ii) suppression of effector T functions such as T cell infiltration and cytotoxicity; iii) genetic mutation and transcriptional suppression of genes with tumor antigen processing and presentation; iv) the changes on the expression of cytokines and chemokines. Strategies designed to overcome immune evasion of immunosuppressive tumors are the focus of therapeutic development in cancer immunotherapy.^[^ ^9^ ^]^

Tumor initiation, progression, and metastasis of PDA are essentially attributable to genetic and epigenetic changes.^[^ ^10^, ^11^ ^]^ Altering the epigenetic programing of histone deacetylases (HDACs), DNA methyltransferases (DNMTs), histone demethylases (HDMs), and associated modulators impacts cancer cell gene transcription and metabolism, immunogenicity of cancer cells or immune cell functionality in the TME.^[^ ^12^, ^13^ ^]^ Immune checkpoint molecules (PD‐1, CTLA‐4, Tim‐3, and LAG‐3), immunogenic tumor‐associated antigens, histocompatibility complex (MHC)‐associated tumor antigen processing/presentation machinery, and immune‐associated genes (GZMB, IFNG, and IL2) are transcriptionally regulated by the epigenetic agents. Thus, epigenetic modulation with ICB‐based therapies has a great potential to improve the efficacy of PDA immunotherapy by reversing the immunosuppressive TME into an effector T cell‐favorable state.^[^ ^14^ ^]^ However, technical advancement of high‐throughput screen approaches to identify such unique therapeutic agents lags behind the clinical development of immune checkpoint inhibitors. As a new tool for drug discovery, tumor organoids recapitulate the TME in structure and function, while 2D monolayer cell culture and tumor cell‐formed spheroids do not preserve the in vivo tumor heterogeneity and diversity.^[^ ^15^, ^16^ ^]^ Therefore, generating PDA tumor organoids may well represent the complex architecture and immunosuppressive TME of the original tumors. Patient‐derived organoids (PDOs) have been developed for human cancers including colon,^[^ ^17^ ^]^ pancreas,^[^ ^18^ ^]^ breast,^[^ ^19^ ^]^ prostate,^[^ ^20^ ^]^ bladder,^[^ ^21^ ^]^ ovarian,^[^ ^22^ ^]^ lung,^[^ ^23^ ^]^ and liver^[^ ^24^ ^]^ cancer, which offer a unique tool for precision medicine. However, few of them were developed to facilitate immunotherapy drug screen and tumor immunology studies.

Based on the functional interaction of breast tumor organoids and tumor‐specific T cells, we recently built up a technical platform for the identification of small molecule inhibitors that potentiate T‐cell‐mediated cytotoxicity.^[^ ^25^ ^]^ While it facilitates high‐throughput drug screen, the breast tumor organoids in this system do not contain infiltrated T cells prior to their co‐culture with T cells, thus do not best reflect the interaction of tumor‐infiltrated T cells with adverse microenvironment inside the tumor. To solve this issue, we have now developed a T cell‐incorporated pancreatic tumor organoid model for high‐throughput drug screen. The pancreatic tumor organoids with defined size and structure were generated from orthotopic pancreatic tumors in mice or surgical tumor tissue samples of patients with pancreatic cancer. The tumor organoids recapitulate the cell composition and immunosuppressive TME of the original tumor and incorporate tumor‐specific T cells from a layer coated on the outside of tumor organoids.

The T cell‐engaging pancreatic tumor organoids are an effective and functional tool to study the infiltration and cytotoxicity of T cells in the tumor, as well as their interaction with cancer cells and TME. The identified epigenetic agents, ITF2357 and I‐BET151, enhanced T cell cytotoxicity, upregulated MHC‐I‐associated antigen presentation of tumor cells, changed immune‐associated gene expression profiles, and modulated TME. The results were further validated using PDA syngeneic mouse models and PDOs. The novelty of our organoid model lies in mimicking the physical barrier of PDA tumor that prevents the tumor‐infiltration of T cells and providing a standardized and well‐reproducible protocol for identifying drug candidates in synergy with ICB for precision cancer immunotherapy.^[^ ^26^ ^]^

## 2 Results

### 2.1 Generation and Characterization of T Cell‐Incorporated PDA Organoids

**Figure 1:**
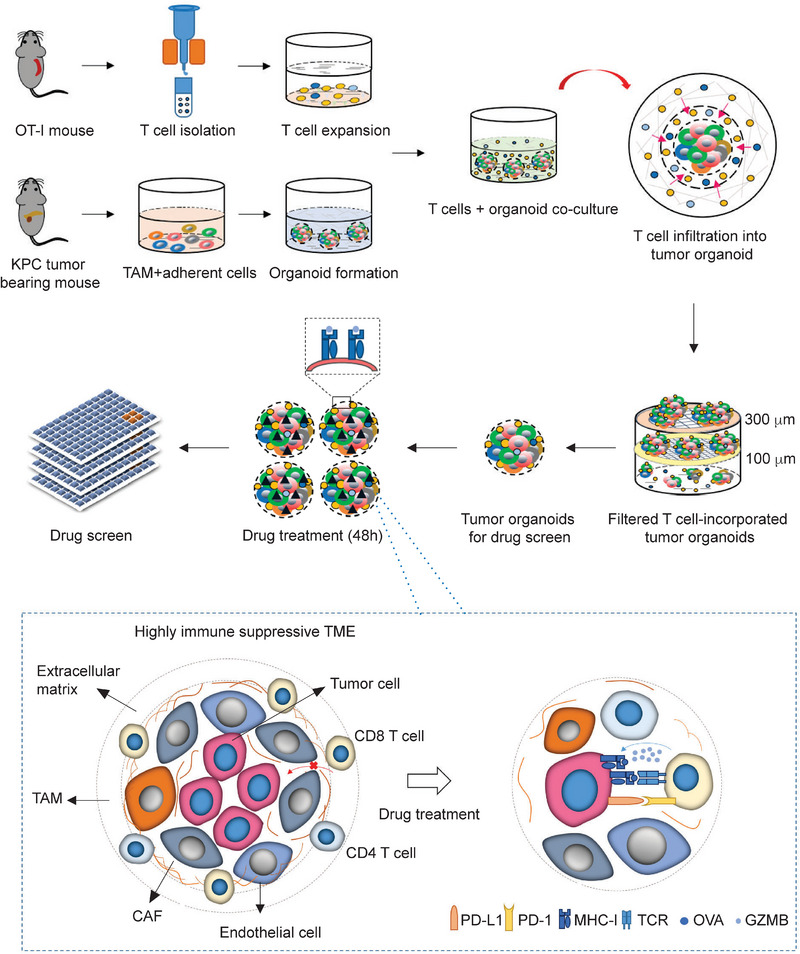
Schematic illustration of a T cell‐incorporated PDA tumor organoid‐based screen for drug discovery. The screen process is composed of pancreatic tumor organoid generation, T cell‐mediated cytotoxicity and drug screen. First, OVA^+^Luc^+^GFP^+^ KPC cells that can express OVA peptide SIINFEKL were orthotopically transplanted to C57BL/6 mice to generate pancreatic tumors. The tumors are harvested and dissociated into single cells. The adherent cells collected at the second day from 2D culture and F4/80 positive cells (macrophages) are combined to generate tumor organoids in 10% Matrigel‐containing culture medium. A total of 4 × 10^5^ cells in 2 mL organoid culture medium were seeded into each well with ultra‐low attachment surface. After 5‐day culture, tumor organoids with diameter between 70 and 150 µm are mixed with CD3/CD28 antibodies pre‐activated and OVA‐specific T cells with a ratio of 1:1000 to 1:2500 in organoid culture medium containing 3% Matrigel and 10 ng mL^−1^ IL2 for 36 h. The T cell‐incorporated tumor organoids with diameter between 100 and 300 µm are collected and treated with each individual drugs for 48 h in the Matrigel‐free medium containing 10 ng mL^−1^ IL2. The tumor cell viability in tumor organoids with and without T cells incorporated was quantified using an Incucyte S3 system.

**Figure 2:**
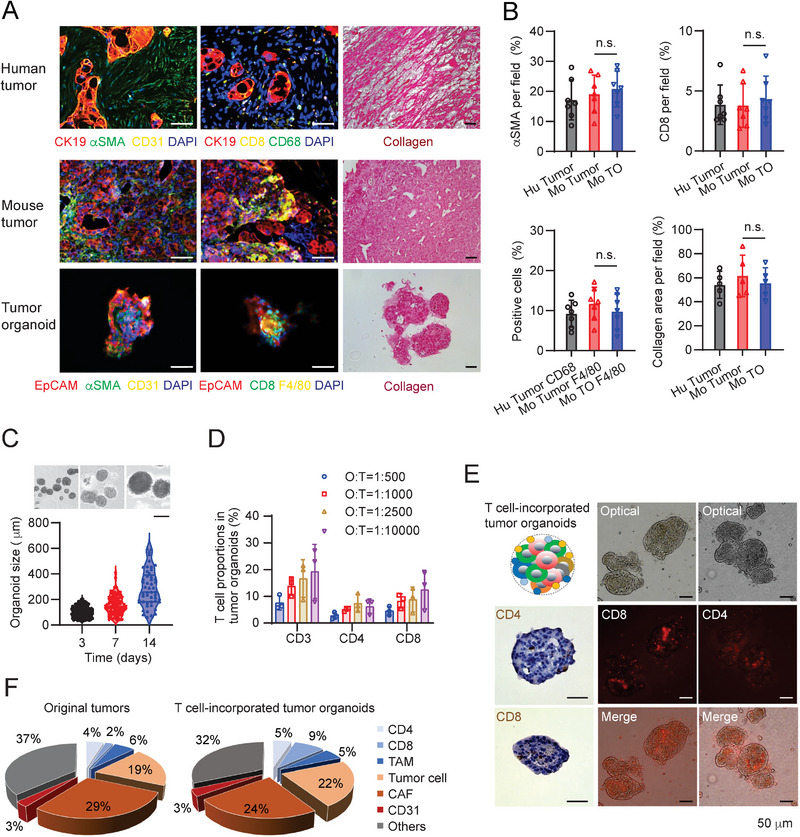
Characterization of mouse PDA tumor organoids. A) Immunofluorescence (IF) and collagen deposition (Picro Sirius red staining) images of human pancreatic cancer samples, mouse KPC tumors and tumor‐derived organoids. CK19 stains for epithelial cells; *α*SMA for cancer‐associated fibroblasts; CD31 for endothelial cells; and CD68 and F4/80 for human and mouse macrophages, respectively. Scale bars = 50 µm. B) The proportions of staining‐positive cells (*α*SMA, CD68, F4/80, or CD8) from IF (*n* = 7, image per group) and Picro Sirius red staining (*n* = 5, image per group) in total cells of the samples as indicated. TO: Tumor organoids. C) Sizes of mouse KPC tumor organoids in culture at day 3, 7, and 14. Optical images showed the corresponding organoid sizes on different days. Scale bars = 200 µm. D) T cell proportions in mouse KPC tumor organoids when packed onto the surfaces of tumor organoids with different Organoid: T cell ratios. The data were collected by flow analysis after the filtered organoids were digested into single cells and stained with T cell antibodies (sample size per group: *n* = 3). Organoid: T cells ratio (O:T). E) Optical, IHC and IF images (CD4, CD8) of T cell‐incorporated tumor organoids. Scale bars = 50 µm. F) Cell composition of mouse KPC orthotopic tumors harvested 30 days post orthotopic injection of KPC cells into C57BL/6 mice, and cell composition of T cell‐incorporated tumor organoids derived from the KPC tumor at the day 7 of organoid culture.

To establish a standard and reproducible protocol for pancreatic tumor organoid platform, we started from pancreatic tumors developed by orthotopic injection of OVA_257‐264_ antigen‐presented KPC (*LSL‐Kras^+/G12D^; LSL‐Trp53^+/R172H^; PDX1‐Cre*) cells into the parenchyma of C57BL/6 mice. The resulting tumors were harvested 30 days post‐injection to generate tumor organoids as described in the Methods. The generated pancreatic tumor organoids reflect the features of the original pancreatic tumor in key components including epithelial tumor cells, vascular endothelial cells, tumor‐associated fibroblasts, and macrophages, the outside Matrigel layer (thickness: ≈25 µm, Figure S1A, Supporting Information) of which was packaged with T cells isolated from OT‐I transgenic mice that recognize the OVA_257‐264_ antigen presented on KPC tumor cells (**Figure** **1**). The T cells in the Matrigel layer gradually infiltrated into the inner tumor organoids in the next 36 h, forming T cell‐incorporated tumor organoids. The cell composition, desmoplasia, immune microenvironment, hypoxia, and culture time of the tumor organoids were determined. In comparison with subcutaneous tumors, the orthotopic KPC tumors are quite different in tumor microenvironment, as indicated in stroma cell components including pancreatic stellate cells (PSCs) and activated cancer‐associated fibroblasts (CAFs)^[^ ^27^ ^]^ stained by glial fibrillary acidic protein (GFAP) and *α*‐smooth muscle actin (*α*‐SMA), respectively (Figure S1B, Supporting Information). *α*‐SMA was a biomarker for cancer associated fibroblasts that contribute to tumor progression by promoting angiogenesis and remodeling the extracellular matrix.^[^ ^28^, ^29^ ^]^ Tumor‐associated fibroblasts and macrophages (human CD68+ or mouse F4/80+) play a major role in the maintenance of immunosuppressive microenvironment in pancreatic cancer (**Figure** **2**A,B).^[^ ^30^, ^31^ ^]^ Particularly, the levels of CAFs in the tumor are negatively correlated with clinical outcomes of patients with PDA (Figure S1C, Supporting Information). Therefore, these two types of cells were packaged in the tumor organoids to create a microenvironment comparable to that of the original tumor.

The collagen deposition secreted by stroma cells formed desmoplasia in the PDA tumor that may prevent immune cell infiltration. Tumor organoids generated from the KPC tumor preserved similar expression levels of collagen as observed in both human and mouse pancreatic tumors (Figure 2A,B). The sizes of the tumor organoids without T cell incorporated were measured at different culture times (Figure 2C). The level of hypoxia in the tumor organoids increased with their growth (Figure S1D, Supporting Information). The oxygen levels ranged from 0.5% to 8% in the tumor organoids with diameter between 100 and 300 µm showed physiologically relevant hypoxia levels of pancreatic cancer as the median oxygen levels ranged from less hypoxic states around 1.2–12.3% to severely hypoxic levels of 0.7%.^[^ ^32^ ^]^ The numbers of T cells incorporated with the tumor organoids were also analyzed by flow cytometry and immunofluorescent staining (Figure 2D,E). Around 7–15% of total T cells were packaged onto tumor organoids when the ratio of tumor organoid versus T cell reached 1:1000 in 3% Matrigel (Figure 2D). To mimic original tumors to the largest extent, the cell composition of the tumor organoids was measured and compared with their original tumors (Figure 2F, Figure S2A, Supporting Information). As macrophages and T cells are the primary immune cells and major contributors to the TME of pancreatic cancer, other CD45^+^ immune cells were excluded from tumor organoids on purpose for a standardized and reproducible protocol (Figure 2F). Although CD8+ cells were only 2% in original tumors, T cells can freely infiltrate to tumor site from blood vessels. The 2% of CD8+ T cell concentration cannot suppress the growth of tumor organoids in vitro. Hence, 9% of CD8+ T cells were coated onto the tumor organoids on purpose, many of which stayed in the Matrigel layer outside of tumor organoids, acting as the external sources like the blood vessel system in tumors. PD‐1, Tim‐3, and Lag‐3 are biomarkers for CD8 T cell exhaustion. Their expression levels in the CD8 T cells from tumor organoids and the original KPC tumors showed no statistical difference (Figure S2B,C, Supporting Information), suggesting that T cell‐incorporated tumor organoids have similar T cell exhaustion effects as in the original tumors. Therefore, the T cells encapsulated in the tumor organoids can be used to assess T cell‐mediated cytotoxicity in vitro that reflects the in vivo activity. The T cell‐incorporated KPC tumor organoids with the diameter of 100–300 µm were next applied for drug screen.

### 2.2 Epigenetic Drug Screen with *α*PD‐1 Based ICB

**Figure 3:**
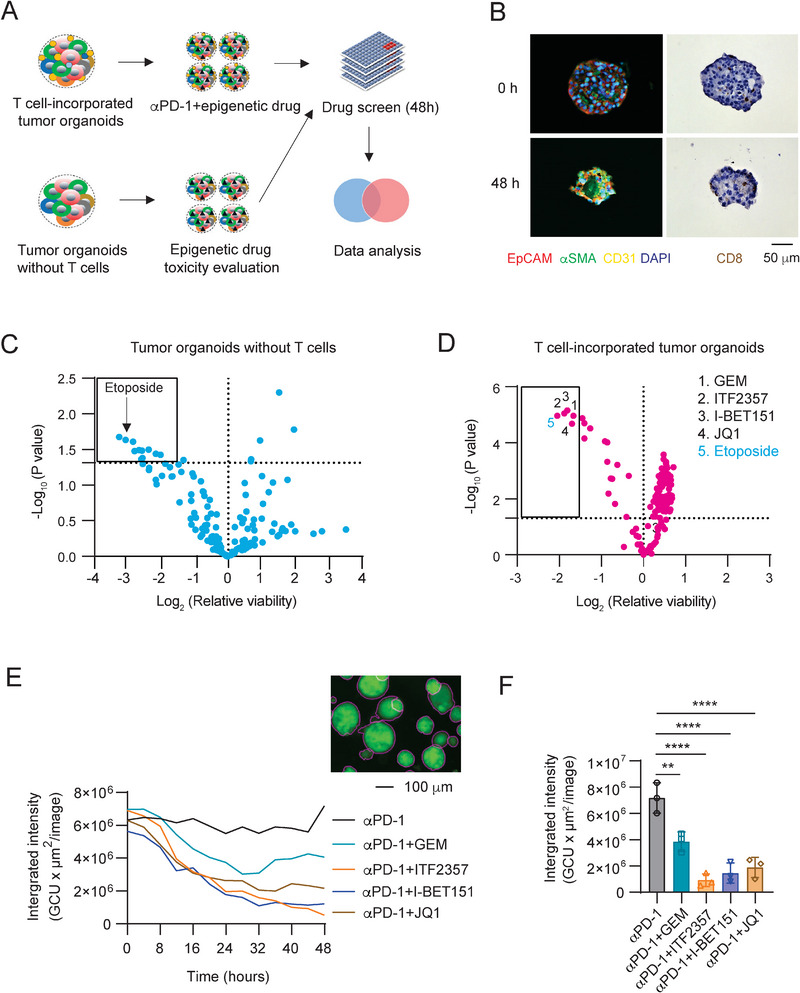
Epigenetic drug screen using mouse PDA tumor organoid platform. A) Schematic illustration of drug screening using 3D tumor organoids with and without T cells incorporated. B) IF and IHC staining of CD8 T cells in the KPC tumor organoids at 0 and 48 h after generation of the T cell‐incorporated organoids. EpCAM, *α*SMA, and CD31 stained cells are shown in red, green, or yellow color, respectively in the IF images. CD8 T cells showed brown color in the IHC images. C) Volcano plot analysis showing the viability of tumor cells in the OVA^+^Luc^+^GFP^+^ KPC tumor organoids treated with vehicle control or compounds (1.0 µm) for 48 h. The compounds within the rectangle had significant toxicity to the tumor organoids (Log_2_[relative viability] < −1.5; *p* < 0.05). D) The viability of tumor cells in the T cell‐incorporated OVA^+^Luc^+^GFP^+^ KPC tumor organoids treated with vehicle control or compounds (1.0 µm) in the presence of *α*PD‐1 (10 µg mL^−1^) for 48 h. The compounds within the rectangle showed significantly enhanced T cell cytotoxicity on cancer cells in the tumor organoids (Log_2_[relative viability]< −1.5; *p* < 0.05). GEM: Gemcitabine. E) The real‐time viability of tumor cells in the OVA^+^Luc^+^GFP^+^ KPC tumor organoids with indicated treatments (*α*PD‐1, GEM + *α*PD‐1, ITF2357 + *α*PD‐1, I‐BET151 + *α*PD‐1, and JQ1 + *α*PD‐1) was monitored and analyzed for 48 h using Incucyte device. The viability of cells was determined by the integrated intensity [mean intensity (CGU) × organoid area (µm^2^)]. F) Histogram analysis of the cell viability for (E) (sample size per group: *n* = 3).

**Figure 4:**
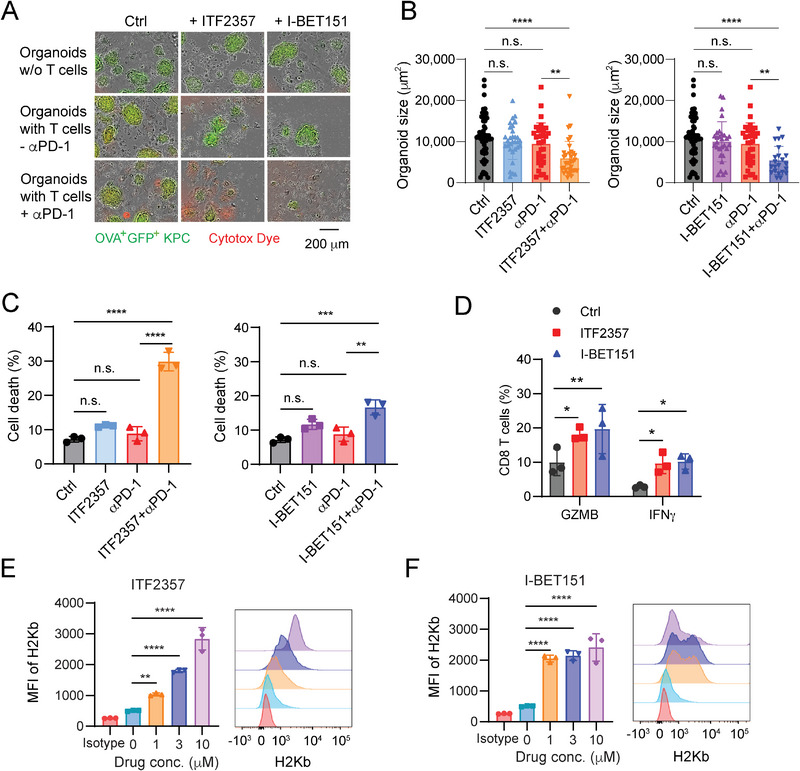
Validation of epigenetic drug candidates using mouse tumor organoids. A) The cytotoxicity of tumor cells in the OVA^+^GFP^+^KPC tumor organoids with and without T cells incorporated upon treatment of isotype antibody control, ITF2357 (1.0 µm), I‐BET151 (1.0 µm), with, or without *α*PD‐1 (10 µg mL^−1^) for 48 h. The images are overlapped with optical light and green/red fluorescence channels. The GFP^+^ KPC tumor cells are shown in green color; red color (Sartorius Cytotox Dye) stains dead cells. Scale bars = 200 µm. B) Quantification of organoid size of the sample groups from (A). The organoid size was analyzed using ImageJ software. Data were presented as mean ± SD by One‐way ANOVA test. **, *p* < 0.01; ****, *p* < 0.0001; n.s., no significance. C) Death rates of the tumor cells in the tumor organoids treated with control, *α*PD‐1, ITF2357, I‐BET151, ITF2357 + *α*PD‐1, or I‐BET151 + *α*PD‐1, were measured by flow cytometry analysis (sample size per group: *n* = 3). Data were presented as mean ± SD by One‐way ANOVA test. **, *p* < 0.01; ***, *p* < 0.001; ****, *p* < 0.0001; n.s., no significance. D) GZMB and IFN*γ* positive cells in the CD8 T cells incorporated in the tumor organoids treated with control or epigenetic compounds (sample size per group: *n* = 3). Data were presented as mean ± SD by Two‐way ANOVA test. *, *p* < 0.05; **, *p* < 0.01. E,F) The mean fluorescence intensity (MFI) of H‐2Kb on the mouse OVA^+^GFP^+^ KPC cells treated with control, ITF2357, or I‐BET151, determined by flow cytometry analysis (sample size per group: *n* = 3). Data were presented as mean ± SD by One‐way ANOVA test. **, *p* < 0.01; ****, *p* < 0.0001.

Epigenetic modulators can impact tumor immunogenicity and microenvironment by regulating transcription of genes associated with tumor cell antigen presentation and responses to immune cells in the tumor, leading to activation of T cells or suppression of immunosuppressive cells such as tumor‐associated macrophages and Treg cells.^[^ ^33^ ^]^ Given the dismal clinical outcome of ICB‐based therapy in the treatment of PDA, a combinational therapy with epigenetic modulation is worthy of investigation for combating immunosuppressive TME and promoting the efficacy of ICB therapy. To this end, we generated T cell‐incorporated tumor organoids to screen epigenetic inhibitors or modulation agents (Table S1, Supporting Information) that can potentiate T cell‐mediated cytotoxicity in the immunosuppressive tumor organoids when used in combination with ICB therapy. The epigenetic inhibitors with a concentration of 1 *µ* m were used to treat the T cell‐incorporated OVA^+^ KPC tumor organoids and the tumor organoids without T cells incorporated were used as a control (**Figure** **3**A). Representative images of the tumor organoids with CD8 T cells incorporated were shown in immunofluorescent and immunohistochemical images (Figure 3B). Compounds (1 *µ* m) with significant toxicity or proliferation‐promoting effect on T cell‐free tumor organoids engaged were excluded for the T cell cytotoxicity focused screen (Figure 3C). The tumor cell viability in organoids as an indicator for the T cell‐mediated cytotoxicity was imaged by the Incucyte S3 System (Essen Bioscience, Inc) (Figure 3D). In the screen, a total of four compounds (Gemcitabine, ITF2357, I‐BET151, and JQ1) exhibited the highest T cell‐mediated cytotoxicity (*p* < 0.05 or −log_10_(*p* value) >1.30; Relative viability < 35% or log_2_(Relative viability) < −1.5) (Figure 3D) in the T cell‐incorporated organoids while no significant cell death was observed in the T cell‐free tumor organoids treated with the same compounds (Figure 3C). The screened drug Etoposide was excluded as it showed high toxicity from the toxicity screen previously (Figure 3C). Gemcitabine (GEM), a chemotherapy drug, was known to promote pancreatic cancer immunotherapy^[^ ^34^ ^]^ and hence it is not our primary candidate for further study. However, screening out this compound proves that the T cell‐incorporated tumor organoid platform is a reliable screening method. The increased T cell‐mediated cytotoxicity in the tumor organoids treated with GEM, ITF2357, I‐BET151, or JQ1 treatment groups in combination with anti‐PD‐1 treatment was shown in time lapse (Figure 3E,F). The results of the three candidates (ITF2357, I‐BET151, or JQ1) were further confirmed by optical/fluorescence imaging (**Figure** **4**A,B and Figure S3A,B, Supporting Information) and flow cytometry results (Figure 4C and Figure S3C,D, Supporting Information). As T cell cytotoxicity indicators, interferon gamma (IFN*γ*),^[^ ^35^ ^]^ and cytolytic granule enzyme Granzyme B (GZMB) had greater expression levels in the CD8 T cells of the tumor organoids when they were treated with ITF2357, I‐BET151, or JQ1 (Figure 4D and Figure S3E,F, Supporting Information). However, treatment of ITF2357, I‐BET151, or JQ1 only on T cells directly isolated from the OT‐I mouse spleen did not increase the secretion levels of IFN*γ*, GZMB, or TNF*α* (Figure S4A,B, Supporting Information), suggesting the drug effects are dependent on the interaction of T cells with the tumor or other stroma cells in the organoids.

To further assess the immune response of the tumor organoids, we examined the drug effect on the antigen presentation of the tumor cells, which is shown by the levels of MHC‐I molecules on the cell surface. We measured the level of H‐2Kb on mouse KPC cells and the level of HLA‐A,B,C on human Panc‐1 cells by flow cytometry when both types of cells were treated with ITF2357, I‐BET151, or JQ1 for 48 h. In all the three drug‐treated groups, H‐2Kb (Figure 4E,F and Figure S4C, Supporting Information) and HLA‐A,B,C (Figure S4D, Supporting Information) were presented at much higher levels compared with their non‐treated control groups. Confocal images also showed that HLA‐A,B,C has higher expression on the tumor cell surface (Figures S4E, Supporting Information). The results indicate that the identified inhibitors (ITF2357, I‐BET151, and JQ1) can boost up the MHC‐I‐mediated antigen presentation on mouse and human pancreatic cancer cells.

### 2.3 Anti‐Tumor Activity of ITF2357 and I‐BET151 In Vivo

**Figure 5:**
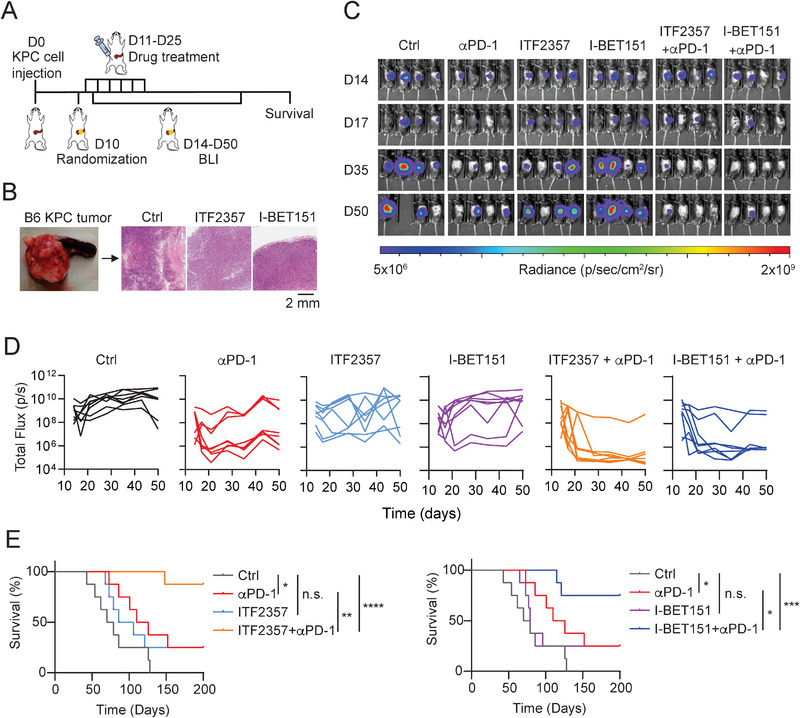
Epigenetic inhibitors augment anti‐tumor activity of immune checkpoint blockade in vivo. A) Drug treatment and bioluminescent imaging scheme of the KPC tumor‐bearing mice. The tumor‐bearing C57BL/6 mice were randomized into six groups with eight mice (4 males +4 females) per group. B) The KPC tumors from the tumor‐bearing C57BL/6 mice and IHC staining images of KPC tumors from control, ITF2357, and I‐BET151 treatment groups. C) Bioluminescent images of KPC tumors from the tumor‐bearing C57BL/6 mice treated with isotype antibody control, *α*PD‐1, ITF2357, I‐BET151, ITF2357 + *α*PD‐1, or I‐BET151 + *α*PD‐1 at day 14, 17, 35, and 50 post orthotopic injection. D) The total flux of KPC tumors from the tumor‐bearing C57BL/6 mice treated with isotype antibody control, *α*PD‐1, ITF2357, I‐BET151, ITF2357 + *α*PD‐1, or I‐BET151 + *α*PD‐1 by bioluminescent Imaging (sample size per group: *n* = 8). E) Survival curves of the KPC tumor‐bearing C57BL/6 mice treated with isotype antibody control, *α*PD‐1, ITF2357, I‐BET151, ITF2357 + *α*PD‐1, or I‐BET151 + *α*PD‐1 combo group. Data were analyzed by the Kaplan–Meier method. *, *p* < 0.05; **, *p* < 0.01; ***, *p* < 0.001; ****, *p* < 0.0001; n.s., no significance.

Among the three compounds identified from our drug screen with tumor organoids, JQ1 is a potent inhibitor of the BET family of bromodomain proteins. It was reported to enhance T cell function in adoptive immunotherapy models^[^ ^36^ ^]^ and anti‐tumor activity in patient‐derived xenograft models.^[^ ^37^ ^]^ These results support the validity of our T cell‐incorporated tumor organoid platform in drug discovery for T cell‐based immunotherapy. Due to its short life in vivo, JQ1 has not been used in human clinical trials. Interestingly, another identified compound, I‐BET151, is a new BET bromodomain inhibitor that inhibits BRD2, BRD3, and BRD4.^[^ ^38^ ^]^ Consistent with the antitumor activity of histone deacetylase (HDAC) inhibitors,^[^ ^39^ ^]^ ITF2357 is a pan‐HDAC inhibitor (also known as givinostat). Next, we wanted to test the anti‐tumor activity of ITF2357 and I‐BET151 in an immunocompetent mouse PDA model in combination with ICB therapy using PD‐1 monoclonal antibody. The mouse KPC cells expressing luciferase (for in vivo tumor imaging) were orthotopically implanted into the pancreas of both male and female C57BL/6 mice and treated with isotype antibody control, *α*PD‐1, ITF2357, I‐BET151, or *α*PD‐1 plus ITF2357 or I‐BET151 combo. The treatment was conducted three times a week for 2 weeks after mice randomization (**Figure** **5**A,B). The tumor growth was monitored by in vivo bioluminescence imaging (Figure 5C). As shown in the results of tumor growth and mouse survival curves (Figures 5D,E), the combo treatment groups showed notably greater suppression on the tumor growth in comparison with the control group as well as single agent treatment group (ITF2357, I‐BET151, or *α*PD‐1). The tumor growth suppression can also be seen in the statistical results at day 43 when no mice died in any groups (Figure S5A,B, Supporting Information). *α*PD‐1 treatment alone showed modest, but statistically significant (*p* < 0.05) anti‐tumor activity compared with the control group. By contrast, addition of ITF2357 or I‐BET151 to the *α*PD‐1 treatment substantially improved the survival of tumor‐bearing mice compared with the *α*PD‐1 treatment alone (*p* < 0.01 for ITF2357 and *p* < 0.05 for I‐BET151). The median survival times (post‐orthotopic injection) from the control, ITF2357, I‐BET151, or *α*PD‐1 alone groups were 70, 86, 79, and 110 days, respectively. The median survival times of the combo groups (ITF2357+*α*PD‐1 or I‐BET151+*α*PD‐1) surpassed 200 days, showing a considerable treatment effect. No overt toxicity was observed in the major organs (heart, lung, kidney, spleen, liver) from the mice treated with ITF2357(20 mg kg^−1^) or I‐BET151 (20 mg kg^−1^) by histological examination (Figure S5C, Supporting Information). Collectively, the results suggest that ITF2357 or I‐BET151 in combination with immune checkpoint inhibitors has a strong translational potential as a therapeutic approach for treating PDA.

### 2.4 Immune Profile Analysis of the Drug Treated KPC Tumors

**Figure 6:**
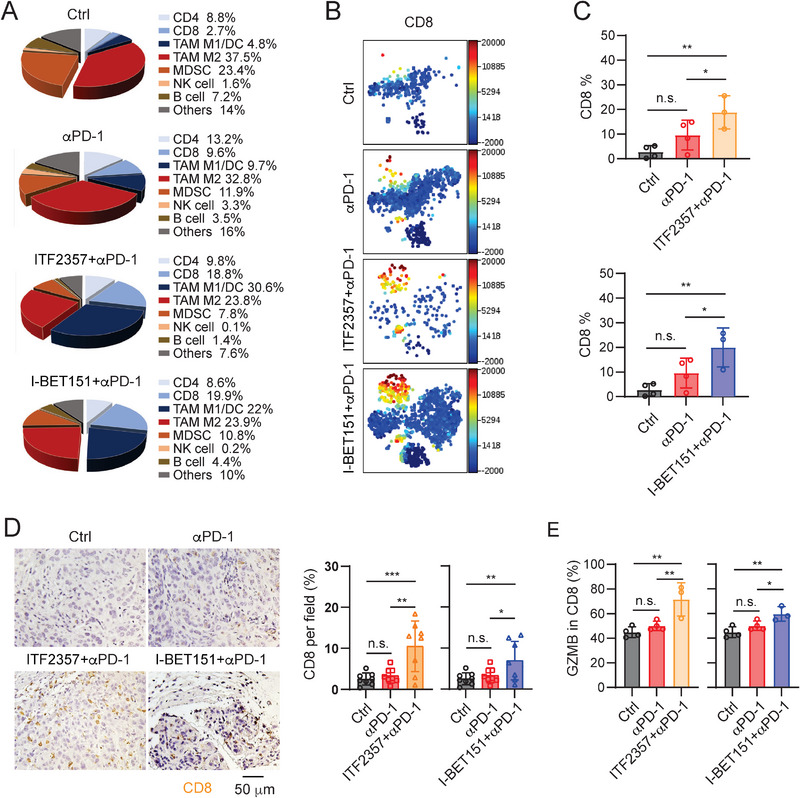
ITF2357 or I‐BET151 in combination with *α*PD‐1 enhances T cell infiltration and cytotoxicity in mouse KPC tumors. A) Pie charts of immune cells in KPC tumors isolated from the KPC‐bearing C57BL/6 mice treated with control, *α*PD‐1, ITF2357 + *α*PD‐1, or I‐BET151 + *α*PD‐1. The control or *α*PD‐1 group has five mice (3 males + 2 females) in each group and both combo treatment groups have six mice (3 males + 3 females) per group. B) t‐distributed stochastic neighbor embedding (t‐SNE) representation of CD8 T positive cells in the KPC tumor‐bearing mice treated with control, *α*PD‐1, ITF2357 + *α*PD‐1, or I‐BET151 + *α*PD‐1. The cell subtypes and proportions in total immune cell populations in the tumors are shown in t‐SNE. The dot plot presenting each channel is spectrum colored and the spectrum value represents the expression intensity of the indicated cell subtype. C) Quantitative analysis of CD8 T positive cells subtypes from t‐SNE results in (B) (sample size per group: *n* = 3 or 4). Data shown as mean ± SD by One‐way ANOVA test. *, *p* < 0.05; **, *p* < 0.01; n.s., no significance. D) IHC images and quantitative results of CD8 T cells in the tumors with indicated treatments (sample size per group: *n* = 8). Data shown as mean ± SD by One‐way ANOVA test. *, *p* < 0.05; **, *p* < 0.01; ***, *p* < 0.001; n.s., no significance. E) GZMB expression levels on CD8 T cells with indicated treatments, measured by Cytek device, and quantified by FlowJo software (sample size per group: *n* = 3 or 4). Data shown as mean ± SD by one‐way ANOVA test. *, *p* < 0.05; **, *p* < 0.01; n.s., no significance.

To understand the roles of ITF2357 or I‐BET151 plus *α*PD‐1 treatment on cancer immunity, we orthotopically injected KPC cells into C57BL/6 mice and treated with isotype antibody control, *α*PD‐1, ITF2357+*α*PD‐1 or I‐BET151+*α*PD‐1 combo group. Tumors were collected at day 30 post injection (Figure S6A) and the tumor weight results (Figure S6B, Supporting Information) confirmed that both ITF2357+*α*PD‐1 and I‐BET151+*α*PD‐1 combo groups displayed statistically lower tumor growth than the control group, consistent with the survival results (Figure 5). The tumors were dissociated into single cells, labelled with the antibody panel (Table S2) and analyzed by the Cytek Aurora instrument. Immune profile analysis (Figure S6C, Supporting Information) of the tumors performed in the Cytobank platform showed that ITF2357+*α*PD‐1 or I‐BET151+*α*PD‐1 combo treatment group had increased percentages of CD8 T cells in the tumors in comparison with the control group or *α*PD‐1 group alone (**Figure** **6**A–C and Figure S6D, Supporting Information). The results were confirmed by the IHC staining of CD8 T cells (Figures 6D). The findings suggest that both ITF2357 and I‐BET151 compounds, in combination with *α*PD‐1 can highly promote T cell infiltration. In both combo groups, CD8 T cells showed higher GZMB expression levels than in the control or *α*PD‐1 treatment group alone (Figure 6E, Figure S6E, Supporting Information), suggesting more cytotoxic CD8 T cells in the combo groups. M1 macrophages are known to have an anti‐tumor activity by producing pro‐inflammatory cytokines, promoting T cell activation, and inducing tumor cell apoptosis.^[^ ^40^ ^]^ The increased number and activation of M1 macrophages (CD11b^+^F4/80^+^Gr1^−^I‐A/I‐E^+^) in response to the drug treatment alone or in combination with ICB could enhance the cytotoxicity of T cells against tumor cells, leading to a more effective anti‐tumor response (Figure 6A, Figures S6D and S7, Supporting Information). Myeloid‐derived suppressor cells (MDSCs: CD11b^+^Gr1^+^) and M2 macrophages (TAM M2: CD11b^+^F4/80^+^Gr1^−^I‐A/I‐E^−^) decreased in the ITF2357+*α*PD‐1 and I‐BET151+*α*PD‐1 combo group in comparison to the control group (Figure 6A, Figures S6D and S7, Supporting Information). However, MDSCs also decreased in the *α*PD‐1 treatment group, suggesting that the effect on MDSCs may be due to the treatment of *α*PD‐1, but not be the treatment of ITF2357 or I‐BET151. The results showed that ITF2357 and I‐BET151 combined with *α*PD‐1 augment T cell infiltration, cytotoxicity, and boost the anti‐tumor activity.

### 2.5 Epigenetic Inhibitors Promote the Antigen Presentation of PDA Cells

**Figure 7:**
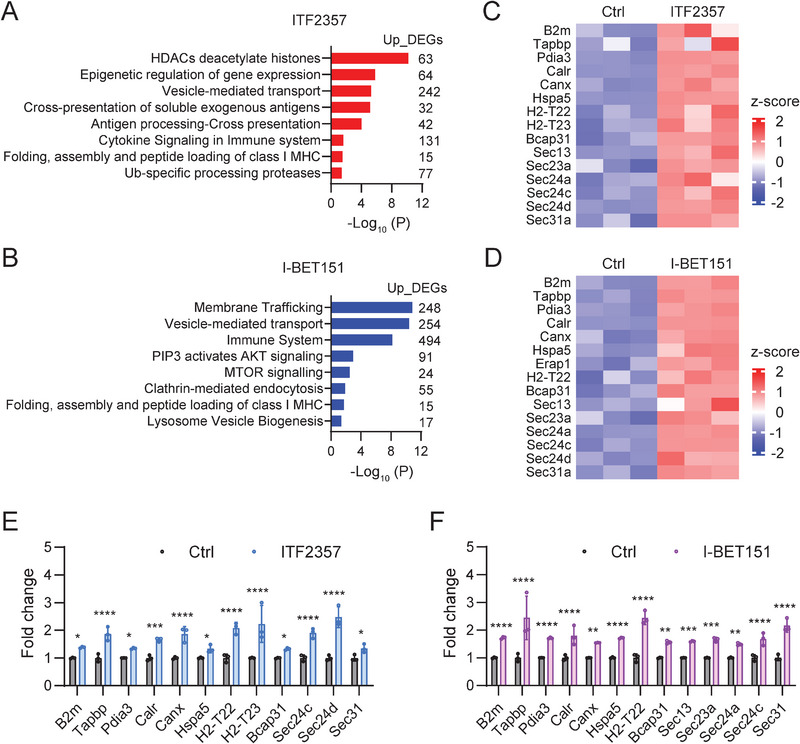
Epigenetic drug treatment up‐regulates antigen processing and presentation of mouse KPC cells. A) Gene pathway analysis of up‐regulated DEGs pathways in the ITF2357 treated KPC cells. B) Gene pathway analysis of up‐regulated DEGs pathways in the I‐BET151 treated KPC cells. C) Heatmap showing the ITF2357‐induced up‐regulation of DEGs enriched in Antigen presentation: folding, assembly, and peptide loading of class I MHC‐I. D) Heatmap showing the I‐BET151‐induced up‐regulation of DEGs enriched in Antigen presentation: folding, assembly and peptide loading of class I MHC‐I. E,F) qPCR validation of up‐regulated genes from (C, D) in mouse KPC cells (sample size per group: *n* = 3). The control group was shared in the two drug treatment conditions. Data were presented as mean ± SD by Two‐way ANOVA test. *, *p* < 0.05; **, *p* < 0.01; ***, *p* < 0.001; ****, *p* < 0.0001.

One of the major reasons that pancreatic ductal adenocarcinoma has limited response to ICB‐based therapy can be ascribed to the genetic mutation and transcriptional suppression of genes associated with tumor antigen processing and presentation. Hence, RNA‐seq analyses performed on single KPC cell line can reveal the genome‐wide changes in gene expression and provide direct evidence how the drug improves the T cell mediated cytotoxicity. To study the molecular mechanism by which ITF2357 and I‐BET151 enhanced the immune response of PDA cells, total RNA‐seq analyses were conducted on the KPC cells treated with or without ITF2357 or I‐BET151 and the gene transcriptomic profiles were assessed (Figure S8A, Supporting Information). Gene ontology (GO) enrichment analyses indicated that treatment of ITF2357, I‐BET151, or JQ1 upregulates genes enriched in the vesicle‐mediated transport pathway and folding, assembly and peptide loading of class I MHC pathway (**Figure** **7**A,B and Figure S8B, Supporting Information). MHC‐I‐associated antigen processing and presentation pathway is the key for tumor cells to be recognized by tumor specific effector T cells, which involves many steps such as protein degradation and processing, vesicle mediated transport and peptide loading on MHC‐I complex.^[^ ^41^, ^42^ ^]^ The differentially expressed genes (DEGs) of the folding, assembly and peptide loading of class I MHC pathway were shown in the heatmaps (Figure 7C,D). Quantitative PCR results (Figure 7E,F) confirmed that most of the DEGs from the KPC cells treated with ITF2357 or I‐BET151 were up‐regulated in comparison with the control group. These results are consistent with the flow cytometry and confocal imaging results showing that ITF2357 or I‐BET151 upregulated H‐2Kb and HLA‐associated antigen presentation in the mouse KPC and human Panc‐1 cells (Figure 4E,F and Figures S4D and S4E, Supporting Information). From the RNA‐seq results, B2M, as the essential component of MHC‐I complex, was also up‐regulated in the KPC cells treated with ITF2357, I‐BET151, or JQ1.

### 2.6 Epigenetic Inhibitors Enhance Anti‐Tumor Activity in PDOs

**Figure 8:**
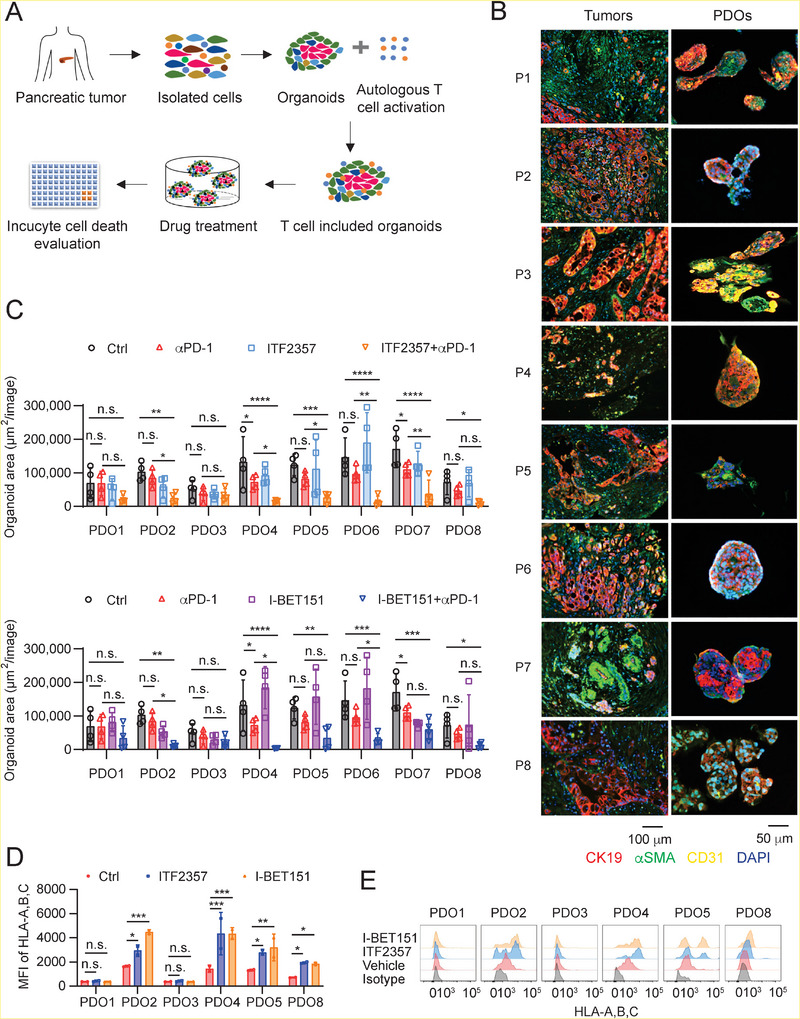
Epigenetic drug treatment enhances the cytotoxicity of autologous T cells in PDOs in combination with ICB. A) Schematic illustration of drug evaluation determined by the cytotoxicity of autologous T cell incorporated in the PDOs. B) Characterization of PDOs from eight pancreatic cancer patients using immunofluorescence analysis. EpCAM^+^, SMA^+^, and CD31^+^ cells represent pancreatic cancer epithelial cells, cancer‐associated fibroblast cells, and endothelial cells, respectively. C) Quantification of T cell‐mediated cytotoxicity in the PDOs treated with control, ITF2357, I‐BET151, *α*PD‐1, ITF2357 + *α*PD‐1, or I‐BET151 + *α*PD‐1 (sample size per group: *n* = 4). Smaller organoid size indicates higher cytotoxicity in the PDOs. The PDOs were treated for 48 h and imaged under the Incucyte device. Control and *α*PD‐1 groups were shared in the two drug treatment conditions. Data were presented as mean ± SD by Two‐way ANOVA test. *, *p* < 0.05; **, *p* < 0.01; ***, *p* < 0.001; ****, *p* < 0.0001; ns, no significance. D) The mean fluorescence intensity (MFI) of HLA‐A,B,C on the Human pancreatic cancer cells treated with control, ITF2357, or I‐BET151 in flow cytometry analysis (sample size per group: *n* = 2). Data were presented as mean ± SD by Two‐way ANOVA test. *, *p* < 0.05; **, *p* < 0.01; ***, *p* < 0.001; ns, no significance. E) Representative flow cytometry data of (D).

Clinical prediction can be made using patient‐derived organoids instead of humanized patient‐derived xenografts (PDXs) to assess the compound treatment effects before the treatment is applied to patients. Here, we developed the standardized protocol using PDOs to assess the therapeutic effect of combinatorial immunotherapy (**Figure** **8**A). In this protocol, the tumor tissue sample from a patient was divided into two parts for autologous T cell expansion and organoid generation, respectively. In vitro expanded and activated T cells were incorporated into tumor organoids within 36 h. The T cell‐incorporated PDOs with size between 100 and 300 µm were treated with 1 *µ* m of ITF2357 or I‐BET151 along with 10 µg mL^−1^ PD‐1 mAb for 48 h and imaged using Incucyte system for cytotoxicity evaluation. The PDOs generated from eight patients with PDA (Table S3, Supporting Information) were confirmed to have similar cell components with their original tumors using immunofluorescence analysis (Figure 8B), in which epithelial cell marker (CK19+), cancer‐associated fibroblast (*α*SMA+) and endothelial cells (CD31+) were characterized as the essential components of tumor tissue. Six out of the eight PDOs (PDO2, PDO4, PDO5, PDO6, PDO7, and PDO8) exhibited markedly higher cancer cell death rates when they were treated with ITF2357, I‐BET151 or JQ1 in combination with PD‐1 mAb, in comparison with that in the control group (Figure 8C and Figure S9A,B, Supporting Information). The PDO2, PDO4, and PDO6 had significantly (*p* < 0.05) greater response to all the combinational treatments of epigenetic inhibitors and *α*PD‐1 than to the treatment of *α*PD‐1 alone. Due to the limited amount of tissue, we were unable to get enough cancer cells for flow cytometry analysis for two samples (PDO6 and PDO7). However, in the 6 PDOs assessed for tumor antigen presentation (indicated by the levels of HLA‐A,B,C on the cell surface), 4 PDOs (PDO2, PDO4, PDO5, and PDO8) exhibited remarkably higher levels of HLA‐A,B,C after treatment with ITF2357 or I‐BET151, while no changes were observed for PDO1 and PDO3 after the treatment due to their extremely low levels of HLA‐A,B,C (Figures 8D,E). The results of the MHC‐I‐associated antigen presentation are well correlated with the results from the T cell cytotoxicity, suggesting that the T cell‐incorporated PDO model is a good platform for potential clinical development.

## 3 Discussion

Pancreatic cancer is often diagnosed at advanced or metastatic stages, and difficult to treat effectively. Patients with metastatic PDA have less than one‐year survival even with optimal chemotherapy.^[^ ^43^ ^]^ Immunotherapeutic agents, particularly checkpoint blockade antibodies, have shown great efficacy against many human cancer types, but almost entirely ineffective against PDA when used as a single agent. The TME of pancreatic cancer drives tumor progression and therapeutic resistance, including immune evasion in ICB‐based therapy. In the TME, there are endothelial cells, fibroblasts, lymphocytes, and myeloid‐derived cells. These cell populations together form an immunosuppressive environment that compromises the antitumor activities of effector T cells. Particularly, pancreatic tumors have dense fibrotic stroma that acts as a physical barrier for immune cell infiltration and TAMs that express negative regulators (such as PD‐L1) against the functionality of T cells.^[^ ^44^, ^45^ ^]^ Modeling the immunosuppressive TME is the key for a 3D organoid platform in drug screen for PDA immunotherapy.^[^ ^46^ ^]^ In this study, the T‐cell‐incorporated organoid model comprises tumor epithelial cells, endothelial cells, tumor‐associated fibroblasts (PSCs and activated fibroblasts), and TAMs. The cell composition well recapitulates what was found in the original tumors. Importantly, intratumor hypoxia gradient in the organoids with size of 100–300 µm is close to physiologically relevant hypoxia state in human pancreatic tumors. We also observed the exhaustion of T cells incorporated in the organoids, indicating that the complex organoid platform developed in this study creates an immunosuppressive environment that photocopies TME in pancreatic cancer. In this organoid culture, Matrigel provides a 3D scaffold for cell attachment and a unique mix of extracellular matrix components, growth factors and cytokines, which can affect cell proliferation, cell differentiation, and angiogenesis.^[^ ^47^ ^]^ It provides a more physiologically relevant microenvironment in 3D cultures that can preserve the structure of tumor tissue.^[^ ^48^ ^]^ On the other hand, the existence of Matrigel in tumor organoids can potentially act as a physical barrier that limits the T cell infiltration and affects the T cell survival, proliferation and activation owing to the presence of growth factors and cytokines.^[^ ^49^ ^]^ Overall, using Matrigel for organoid culture not only can provide a 3D scaffold biomechanical matrix but also create a more realistic biochemical environment to model the spatial and functional relationships among different cell types.^[^ ^50^ ^]^

Tumor organoid models, especially PDOs, are one of the major driving forces that promote the revolutionization of precision medicine. The ability of organoid models to preserve genetic and physiological features of the original tumor offers unprecedented opportunities for cancer drug screen, preclinical validation, and clinical prediction and evaluation. PDOs are often more heterogeneous, with a variety of tumor antigens and immune evasion mechanisms that can limit the T cell‐mediated cytotoxicity.^[^ ^48^ ^]^ The human PDO model could provide a more representative and clinically relevant model for immunotherapy approach and a better therapeutic option for human patients. However, human sample sources are often limited and current techniques for human organoid models are often uncontrolled and irreproducible due to cancer tissue sampling, processing, and culturing procedures and conditions, which can increase the difficulty of using PDOs for T cell‐mediated cytotoxicity assay as a standard protocol. While the mouse OVA+ KPC tumor organoids provide a single antigenic epitope from OVA+ expressing tumor cells that can be targeted by certain population of T cells, it provides a more controlled and standardized system for studying antigen‐specific T cell responses. Also, it serves as a reproducible antigen system with sufficient sample sources that can be used to compare different experimental conditions and treatment outcomes.

To accurately recapitulate the intra‐ and inter‐ tumoral heterogeneity and eliminate the undesirable technical variability in cancer organoid generation and culture, it is of great importance to establish a reliable and standardized platform that rapidly moves translatable insights into patient care. We recently reported a high‐throughput drug screen platform based on the functional interaction of breast tumor organoids and tumor‐specific cytotoxic T cells, in which cell composition, size, and hypoxia of tumor organoids were characterized and optimized for immunotherapy drug screen.^[^ ^25^ ^]^ However, this tumor organoid model did not contain immune cells and thus could only be used to study the immune response of tumor cells when they are co‐cultured with tumor‐specific T cells. In this study, we generated an improved organoid model that includes tumor‐associated macrophages and tumor‐specific T cells in the organoid. This model has defined tumor features, cell components, fully recapitulated immunosuppressive TME that engages T cells, enabling high‐throughput drug screen and prediction of clinical responses of pancreatic cancer immunotherapy.

The T cell‐incorporated tumor organoids in this study create physiologically relevant and immunosuppressive TME by maintaining the complex cell components and structures, where cancer‐associated fibroblasts and secreted collagens as T cell infiltration barriers were well preserved. TAMs (F4/80^+^CD11b^+^) and Tregs cells in organoids were also preserved with similar proportions as in original tumors. Although they are not the essential components of cancer organoids,^[^ ^51^, ^52^, ^53^ ^]^ T cells and TAMs are the main components that often determine the response to immunotherapy in pancreatic tumors.^[^ ^54^ ^]^ In preparing tumor organoids, T cells were first packaged in the border ring region of the organoid core, reflecting the low infiltration of immune cells and stroma barriers in the immunosuppressive environment observed in human pancreatic tumors.^[^ ^55^ ^]^ Tumor organoids with greater size have increased cell death in the core area due to hypoxia inside the organoids. Hypoxia is a crucial factor to be considered for organoid models as hypoxia signaling also triggers resistance to cancer therapy.^[^ ^56^ ^]^ In this study, we selected the T cell‐incorporated tumor organoids with size between 100 and 300 µm in diameter, which exhibited physiologically relevant hypoxia. Uniform sizes of tumor organoids help to generate reproducible and reliable results in drug screen and validation.

Reinvigorating the immune response of cancer cells is equally important as activating antitumor immune cells in cancer immunotherapy. Here we attempted to identify epigenetic inhibitors that potentiate PD‐1‐based immune checkpoint blockade by reprogramming the transcription of pancreatic cancer cells. One identified compound ITF2357 is a potent HDAC inhibitor that was reported to have antitumor activity.^[^ ^57^ ^]^ The roles of HDAC inhibitors in anti‐tumor immunity have been emerging, including the upregulation of genes involved in the antigen presentation and costimulatory molecule expression in cancer cells, and altering the activity of immune cells via cytokine and chemokine secretion.^[^ ^58^ ^]^ The development of potent inhibitors of BET proteins, which target bromodomains, has been a feasible approach to suppress oncogenic networks within tumors. Both I‐BET151 and JQ1 are BET inhibitors.^[^ ^36^, ^59^ ^]^ In particular, JQ1 in combination with PD‐1 blockade, was reported to enhance anti‐tumor immunity in colorectal cancer.^[^ ^60^ ^]^ We studied potential molecular mechanisms underlying immune relevant functions of these epigenetic inhibitors. Interestingly, all the three inhibitors showed little effect on the activity of T cells without co‐culture with tumor cells or tumor organoids, indicating that the anti‐tumor activity may be attributed to their effects on cancer cells or tumor microenvironment. The reactome pathway enrichment analysis of RNA‐seq data reveals significant changes in the expression of the genes involved in the immune‐related signaling pathways. Particularly, all the three compounds upregulated the gene expression in the MHC‐I pathway, which led us to study the MHC‐I‐mediated antigen presentation of pancreatic cancer cells. In addition to its upregulation of antigen processing and presentation, JQ1 also promoted Interleukin‐2 (IL2) family signaling, interferon‐*γ* (IFNG) signaling, and cytokine signaling in cancer cells. Both ITF2357 and I‐BET151 showed great therapeutic outcomes in syngeneic mouse models and PDO models when combined with PD‐1 blockade, suggesting their strong potential for clinical development in PDA therapy.

## 4 Conclusion

In this study, we built a standardized platform of T cell‐engaging pancreatic tumor organoids for high‐throughput drug screen and precision therapy. This organoid model allows the investigation of T cell infiltration and cytotoxicity on tumor cells within immunosuppressive microenvironment that recapitulates the desmoplastic and suppressive pancreatic tumor microenvironment. The tumor organoids mimic the immune infiltration of T cells through the physical barrier of PDA tumor and preserve the cell composition, histological structure, hypoxia as well as immune‐cancer interaction as seen in the original tumor, which will accelerate discovery of actionable epigenetic drugs in combination with ICB‐based therapy for PDA and create new precision immunotherapies in the future.

## 5 Experimental Section

### Cell Lines, Primary Cancer Samples, and Mice

Mouse pancreatic GFP^+^Luc^+^ KPC cell line was obtained from Dr. Ronald A. DePinho (MD Anderson Cancer Center, Houston, Texas, USA). The cell line was transfected with pLVX‐IRES‐Neo‐ full‐length OVA plasmid to generate OVA^+^ Luc^+^GFP^+^ KPC cell line. Human Pancreatic ductal adenocarcinoma PANC‐1 cell line was purchased from ATCC. Both cell types were maintained in Dulbecco's modified eagle medium (DMEM) containing 10% fetal bovine serum (FBS) and 1% penicillin/streptomycin (HyClone). Mouse PDA tumor sample cells were culture in DMEM/F12 culture medium supplemented with 10% FBS, 2 mm Ultraglutamine I and 1% penicillin/streptomycin. Mouse T cells were cultured in the PDA tumor cell culture medium supplemented with 10 ng mL^−1^ mouse IL2 (BioLegend, 575402). Human pancreatic cancer tissue samples in this study were provided by the Tissue Procurement and Distribution Core of Indiana University Simon Cancer Center (Table S3, Supporting Information). The cells from pancreatic cancer patients were maintained in DMEM/F12 culture medium containing 10% HBS, 1% penicillin/streptomycin. Human T cells were cultured in the culture medium for human PDA cancer cells with extra 2000 IU mL^−1^ human IL2 (Peprotech, 200‐02).

C57BL/6 mice and OT‐I mice were purchased from the Jackson Laboratory and housed in the animal facility of Indiana University. The transgenic T cell receptor from OT‐I mice recognizes the ovalbumin peptide SIINFEKL (OVA_257–264_) on H‐2Kb presented by mouse tumor cells.

### Tumor Dissociation and Cell Isolation

Single‐cell suspensions from mouse and human pancreatic cancer tissues were obtained using the tumor isolation kits (Miltenyi Biotec, mouse,130‐096‐730; human, 130‐095‐929;) and the gentle MACS Dissociator (Miltenyi Biotec, 130‐093‐235) after the tumors were cut into small pieces with diameter around 2–4 mm. T cells from the spleen of OT‐I mice were enriched using the mouse Pan T cell isolation kit II (Miltenyi Biotec, 130‐095‐130) after spleens were minsed by a 3‐mL syringe over a cell strainer with 40‐µm pore size (Fisher Scientific, 22‐363‐547) and lysed by RBC Lysis Buffer (BioLegend, 420301). Autologous T cells from human pancreatic tumor samples were enriched using the human Pan T cell isolation kit (Miltenyi Biotec, 130‐096‐535) after tumor tissues were digested into single cells by the tumor isolation kits (Miltenyi Biotec, 130‐095‐929). Mouse tumor‐associated macrophages were isolated from mouse cell suspensions using the mouse anti‐F4/80 MicroBeads (Miltenyi Biotec, 130‐110‐443).

### Pancreatic Tumor Organoid Generation

The KPC‐derived tumors (tumor volume ≈ 200–400 mm^3^) developed from orthotopic transplantation in C57BL/6 mice were harvested, dissociated, and plated onto 100 mm tissue culture dish with DMEM/F12 supplemented with 10% FBS and 1% penicillin/streptomycin, and cultured at 37 °C with 5% CO2 overnight. Suspended tumor‐associated macrophages (TAMs) were collected using the mouse anti‐F4/80 microbeads (Miltenyi Biotec,130‐110‐443). The adherent cells on the culture dish were trypsinized, collected, and mixed with the enriched TAMs. The mixed cells were pipetted into cold mouse pancreatic cancer culture medium^[^ ^61^ ^]^ (Table S4, Supporting Information) containing 10% Matrigel (Corning) at concentration of 2 × 10^5^ cells mL^−1^. The cell suspension was immediately transferred at 2 mL well^−1^ to the 37 °C pre‐warmed six‐well microplate with ultra‐low attachment surface (Corning, 3471). Organoids without T cells were generated from the mixed cells within one week to maintain the cell composition of the original tumor as tumor cells can outgrow other cell types in long‐term culture.^[^ ^25^ ^]^ The incorporated CD4 and CD8 T cell populations were measured by flow cytometry before use. The organoids were collected using cell strainers with pore size between 70 and 150 µm and then packaged with anti‐CD3/anti‐CD28 activated T cells with a ratio of 1:1000 to 1:2500 in the cold culture medium containing 3% Matrigel and cultured in the 37 °C pre‐warmed six‐well microplate with ultra‐low attachment surface for 36 h to generate T cell‐incorporated tumor organoids. Matrigel can provide a more physiologically relevant cell environment in 3D cultures. Tumor cells cultured in the Matrigel are more closely to their real behaviors in vivo than in the common 2D culture environment.^[^ ^50^ ^]^ The T cells aggregated in the Matrigel surrounding pancreatic tumor organoid and penetrated into the organoids. Tumor organoid collection and cytotoxicity experiment should be performed at 37 °C to maintain solidification of Matrigel, with purpose to hold T cells firmly with tumor organoids. Finally, T cell‐incorporated tumor organoids with diameter between 100 and 300 µm were used for cytotoxicity and drug screen experiment. Patient‐derived organoids (PDOs) were generated following the similar procedures as the mouse KPC‐derived tumor organoids where the culture medium was replaced with human pancreatic cancer organoid culture medium.^[^ ^19^ ^]^

### T Cell Cytotoxicity and Epigenetic Drug Screen

In the T cell‐mediated cytotoxicity experiment, we constructed a mouse OVA^+^Luc^+^GFP^+^ KPC cell line that presents OVA_257‐264_ antigen. The antigen can be recognized by T cells from OT‐I mouse due to the transgenic inserts of Tcra‐V2 and Tcrb‐V5 genes. Autologous T cells were isolated from human pancreatic cancer tissues and expanded in the DMEM/F12 medium containing 10% HBS, 2 mm Ultraglutamine I, 1% penicillin/streptomycin and 2000 IU mL^−1^ human IL2 for one week. T cells cannot be fully activated and undergo cytotoxicity when co‐cultured with PDOs lacking immune cells (such as dendritic cells) and co‐stimulatory molecules.^[^ ^62^ ^]^ The high diversity in the activation and dysfunctional states of autologous T cells could also increase difficulty in establishing a standard cytotoxicity assay.^[^ ^63^ ^]^ Hence, T cells were activated with anti‐CD3 and anti‐CD28 before use for mouse and human tumor organoid model. We stimulated T cells for 48 h with the anti‐CD3 (BioLegend, mouse, 100238; human, 300438) and anti‐CD28 antibodies (BioLegend, mouse, 102116; human, 302934) pre‐coated on 24‐well microplates (5 µg mL^−1^, overnight). The T cell‐incorporated organoids with diameter between 100 and 300 µm were collected in organoid culture medium in the microplates with ultra‐low attachment surface. In drug screen, OVA^+^Luc^+^GFP^+^ KPC tumor organoids with and without T cell incorporated were seeded at 40 organoids per well in 384‐well microplate with low attachment surface and treated with 1 *µ* m of individual epigenetic compounds in the epigenetic compound library (Cayman chemical, 11071) for 48 h.^[^ ^25^ ^]^ The mouse KPC tumor cell viability in tumor organoids with and without T cells incorporated was indicated by GFP fluorescence levels measured by an Incucyte S3 system (Essen Bioscience, Inc). Human PDOs were treated with isotype control, 1 *µ* m of individual epigenetic compounds, or compounds with *α*PD‐1, and stained with the Cytolight Rapid Green dye (Sartorius, 4705) for live cell labeling before transferring into the Incucyte S3 system for cell viability determination for 48 h. The fluorescence intensity and fluorescence area of tumor organoids representing the tumor cell viability from every image were quantified by the software integrated in the Incucyte device.

### Hypoxia Assay

Hypoxia of tumor organoids was measured using the Image‐iT Red Hypoxia reagent (Invitrogen). Experimental protocol follows the instructions of the product. Images were taken under a Leica DM4B microscope. The mean fluorescence intensity (MFI) from the images representing hypoxia in organoids was determined by the ImageJ software 1.50e.

### T Cell Function Analysis

After drug treatment, IFN*γ* and GZMB expression levels in CD8 T cells incorporated in tumor organoids were detected. Tumor organoids were digested into single cells by TrypLE Express at 37 °C shaking at 300 rpm for 15 min. The single cells including CD8 T cells were stimulated with 50 ng mL^−1^ phorbol 12‐myristate 13‐acetate (PMA) (Sigma‐Aldrich) and 1 *µ* m ionomycin (Sigma‐Aldrich) for 4 h, in the presence of 5 µg mL^−1^ Brefeldin A, which can amplify intracellular cytokine staining signals.^[^ ^64^ ^]^ The cells were stained with APC/Cy7‐conjugated anti‐CD8*α* (BioLegend) and later PerCP/Cy7‐conjugated anti‐GZMB and APC‐conjugated anti‐IFN*γ* (BioLegend) for intracellular staining. The stained cells were analyzed by flow cytometry.

### Immunohistochemistry

Tumor organoids were embedded in 10% Histogel (Fisher scientific, 22‐110‐678) following the protocol from the product and then embedded in paraffin blocks. Immunohistochemistry (IHC) staining for tumors and organoid samples was performed using the protocol mentioned.^[^ ^65^ ^]^ The primary antibodies used in this experiment include anti‐*α*‐Smooth Muscle Actin (*α*‐SMA) (Cell Signaling Technology, clone: D4K9N), anti‐glial fibrillary acidic protein (GFAP) (Cell Signaling Technology, Clone GA5, 3670), and anti‐CD8*α* (Cell Signaling Technology, clone: D4W2Z).

### Immunofluorescence

The Immunofluorescence (IF) staining procedure on tissue slides from paraffin blocks follows the protocol described previously,^[^ ^25^ ^]^ The primary antibodies used include anti‐EpCAM (Abcam, ab32392), anti‐Cytokeratin 19 (CK19) (Abcam, clone EP1580Y, ab52625), FITC‐conjugated anti‐*α*‐SMA (GeneTex, GTX72531), Spark YG 570‐conjugated anti‐mouse CD31 (BioLegend, 102531), FITC anti‐mouse CD3 Antibody (BioLegend,100204), Spark YG 570 anti‐mouse F4/80 (123159), PE‐conjugated anti‐human CD31 (BioLegend, 303106). Samples stained with anti‐EpCAM and anti‐CK19 primary antibodies were labeled with secondary antibody Alexa Fluor 647‐conjugated anti‐rabbit IgG (BioLegend, 406414). Images were taken by a Leica DM4B fluorescent microscope or a confocal microscope.

### Animal Studies

The protocols for the mouse experiments were approved by the Animal Care and Use Committee of Indiana University School of Medicine. Male and female C57BL/6 mice with 6‐week‐old were performed with orthotopic injection. 2 × 10^5^ Luc^+^GFP^+^ KPC cells in a volume of 50 *µ*L PBS buffer were injected into the pancreas after the shaved mice were anesthetized and a small incision of the skin and muscle layer were cut to expose the pancreas. The wound was stitched up with surgical sutures and would clips after cell injection. In the survival experiment, the tumor‐bearing mice were randomized into different groups based on the luciferase data from the IVIS Spectrum In Vivo Imaging System (PerkinElmer) at day 10. The mice with eight mice per group (4 males + 4 females) were treated with isotype antibody control (Bioxcell, rat IgG2a, 10 mg kg^−1^), *α*PD‐1 (10 mg kg^−1^), ITF2357 (20 mg kg^−1^), I‐BET151 (20 mg kg^−1^), ITF2357 (20 mg kg^−1^) + *α*PD‐1 (10 mg kg^−1^) or I‐BET151 (20 mg kg^−1^) + *α*PD‐1 (10 mg kg^−1^) three times a week for 2 weeks. The mice were imaged by the IVIS Spectrum In Vivo Imaging System every week for 2 months.

To analyze the immune profiling changes in the KPC tumor‐bearing mice treated with isotype control, *α*PD‐1, ITF2357 + *α*PD‐1 or I‐BET151 + *α*PD‐1, 2 × 10^5^ Luc^+^GFP^+^ KPC cells were orthotopically injected into the pancreas with same protocol as in the above survival experiment. The mice were treated with isotype control (Bioxcell, rat IgG2a, 10 mg kg^−1^), *α*PD‐1 (10 mg kg^−1^), ITF2357 (20 mg kg^−1^) + *α*PD‐1 (10 mg kg^−1^) or I‐BET151 (20 mg kg^−1^) + *α*PD‐1 (10 mg kg^−1^). ITF2357 and I‐BET151 were used three times a week for 2 weeks and *α*PD‐1 were used two times a week for 2 weeks. The control or *α*PD‐1 group has five mice (3 males + 2 females) and both combo treatment groups have six mice (3 males + 3 females). Tumors were harvested at day 30 post injection and analyzed by the Cytek Aurora system.

### Flow Cytometry

Cell sample acquisition was performed using LSR Fortessa (BD Biosciences). SYTOX Blue (BioLegend, 425305, Dilution 1:100) or Fixable Viability Dye eFluor 506 (Invitrogen, 65‐0866‐14) were used to stain dead cells. The BD cytofix/cytoperm fixation permeabilization kit (BD Biosciences, BD 554714) was used to fix cells for intracellular cell staining. The PBS buffer containing 0.5% BSA and 6% Goat serum was used to dilute antibodies. The dilution buffer mixed with Trustain FcX antibody (BioLegend, mouse:156603; human:422301, Dilution 1:1000) was used for cell blocking. The antibodies used in flow cytometry are listed in Table S5, Supporting Information. The compensation was performed using the anti‐rat and anti‐hamster Ig *κ*/Negative Control Compensation Particles Set (BD Biosciences, 552845) or anti‐mouse Ig *κ*/Negative Control Compensation Particles Set (BD Biosciences, 552843). Doublet exclusion was performed in data analysis. No less than ten thousand events per sample were acquired in flow cytometry. Data analysis was conducted using FlowJo software (version10.6.0).

### Confocal Microscopy

Human Panc‐1 cells were seeded with a concentration of 1 × 10^5^ cells well^−1^ in the four‐well slide (Millicell EZ slide, PEZGS0416) and treated with 1 µm ITF2357 or I‐BET151 for 48 h. The cells were stained with Alexa Fluor 647 conjugated anti‐human HLA‐A,B,C antibody (BioLegend, 311414, dilution 1:100) for 2 h and DAPI (4',6‐Diamidino‐2‐Phenylindole, Dihydrochloride) (Sigma‐Aldrich, D1306, dilution 1:1000) for 5 min. The slides were mounted with ProLong Gold anti‐fade Mountant (Thermo Fisher, P36930). Images were captured under the Leica TCS SP8 confocal imaging system and analyzed by the Imaris x64 8.1.2 software.

### Cytek Aurora Experiment

The Luc^+^GFP^+^ KPC tumors at day 30 post‐injection were isolated from C57BL/6 mice and dissociated into single cells. The cells were stimulated with 50 ng mL^−1^ phorbol‐12‐myristate‐13‐acetate (PMA) and 1 µm Ionomycin for 4 h in the presence of 5 µg mL^−1^ brefeldin A. The cells were stained with the antibody panel (Table S2, Supporting Information). The *α*‐SMA, GZMB, IFN*γ*, and TNF*α* antibodies from the panel were stained after cell fixation. 4 × 10^5^ single cells from each sample were acquired in Cytek Aurora (Biosciences). The data were analyzed using the FlowJo software (version10.6.0) or the Cytobank platform.^[^ ^66^ ^]^

### Quantitative RT‐PCR

The miRNeasy Mini kit (Qiagen, 157‐029‐493) was used to isolate total RNA from the mouse KPC cells treated with vehicle control, ITF2357, or I‐BET151 for 48 h. The RNA samples were reverse transcribed into cDNA using the qscriptXLT cDNA superMix reagent (Quantabio). Quantitative PCR was conducted using a Quantstudio 6 Flex system (ThermoFisher Scientific). The PCR primer sequences are included in Table S6, Supporting Information.

### RNA Sequencing

Total RNA was extracted from the Luc^+^GFP^+^ KPC cells using miRNeasy Mini kit (Qiagen). The QIAseq FastSelect rRNA Removal HMR Kit (Qiagen) was used to remove ribosomal RNA and the KAPA RNA Hyper Prep Kit (Roche Corporate) was used to construct the cDNA library. The library pool was sequenced in 100b paired end read format using the NovaSeq 6000 system (Illumina) at the Center for Medical Genomics, Indiana University School of Medicine (IUSM).

### RNA‐seq Analysis

The analysis of RNA‐seq data followed the procedures described previously.^[^ ^25^ ^]^ The differential expressed genes (DEGs) were determined by a false discovery rate (FDR) with cut‐off of 0.05 and visualized with volcano plot using MATLAB software. The Database for Annotation, Visualization, and Integrated Discovery (DAVID) functional analysis^[^ ^67^ ^]^ was used to analyze the gene enrichment analysis on DEGs from Gene Ontology (GO) database and Kyoto Encyclopedia of Genes and Genomes (KEGG) database. The reactome pathway enrichment analysis and the heatmaps for DEGs from the folding, assembly and peptide loading of class I MHC pathways were plotted using the GraphPad Prism 9 software.

### Quantification and Statistical Analysis

Flow cytometry data were analyzed using FlowJo software (version 10.6.0). The sizes of tumor organoids taken under optical microscope were quantified by the ImageJ software (1.50e). The Cytek results were analyzed by the Cytobank platform. The confocal images were analyzed by the IMARIS software (version 8.1.2). The histological images of the organs of C57BL/6 mice were evaluated by the Aperio ImageScope software. The sample size (*n*) was indicated in the figure legend of each experiment or in the methods section. Data were analyzed to generate graphs using GraphPad Prism 9 software. Statistical analysis was performed using one‐way ANOVA or two‐way ANOVA by the GraphPad Prism 9 software as needed. All the data are indicated as mean ± SD. *p* value indicated as ∗ (*p* < 0.05), ∗∗ (*p* < 0.01), ∗∗∗ (*p* < 0.001) and ∗∗∗∗ (*p* < 0.0001). *p* value lower than 0.05 is considered significantly different.

## Conflict of Interest

M.O. received research funds from Eli Lilly and Bayer and has consulting roles with Pfizer, AstraZeneca, and Novartis. X.L. is a cofounder of Reactimm Therapeutics LLC. X.L. is an inventor on the pending patent PCT/US2021/062842, “Methods to sensitize cancer cells and standardized assay for immunotherapy”.

## Supporting information

Supporting Information

## Data Availability

The data that support the findings of this study are available from the corresponding author upon reasonable request.
