# Supplementary material for: A T Cell‐Engaging Tumor Organoid Platform for Pancreatic Cancer Immunotherapy
Source: Adv Sci (Weinh). 2023 Jun 4;10(23):2300548. doi: 10.1002/advs.202300548 (PMC10427404; doi:10.1002/advs.202300548)
Supplement: Supplementary file 1 — Supporting Information [file ADVS-10-2300548-s001.pdf]

ADVANCED SCIENCE
Open Access

Supporting Information

for *Adv. Sci.*, DOI 10.1002/advs.202300548

# A T Cell-Engaging Tumor Organoid Platform for Pancreatic Cancer Immunotherapy

*Zhuolong Zhou, Kevin Van der Jeught, Yujing Li, Samantha Sharma, Tao Yu, Ishara Moulana, Sheng Liu, Jun Wan, Paul R. Territo, Mateusz Opyrchal, Xinna Zhang*, Guohui Wan* and Xiongbin Lu**

## Supplementary Information

# A T cell-engaging Tumor Organoid Platform for Pancreatic Cancer Immunotherapy

*Zhuolong Zhou, Kevin Van der Jeught, Yujing Li, Samantha Sharma, Tao Yu, Ishara Moulana, Sheng Liu, Jun Wan, Paul R. Territo, Mateusz Opyrchal, Xinna Zhang, Guohui Wan, Xiongbin Lu*

## Inventory of Supplementary Information

- **Supplementary Figure 1-9 and Figure Legends**
- **Supplementary Table 1-6**

# Fig. S1

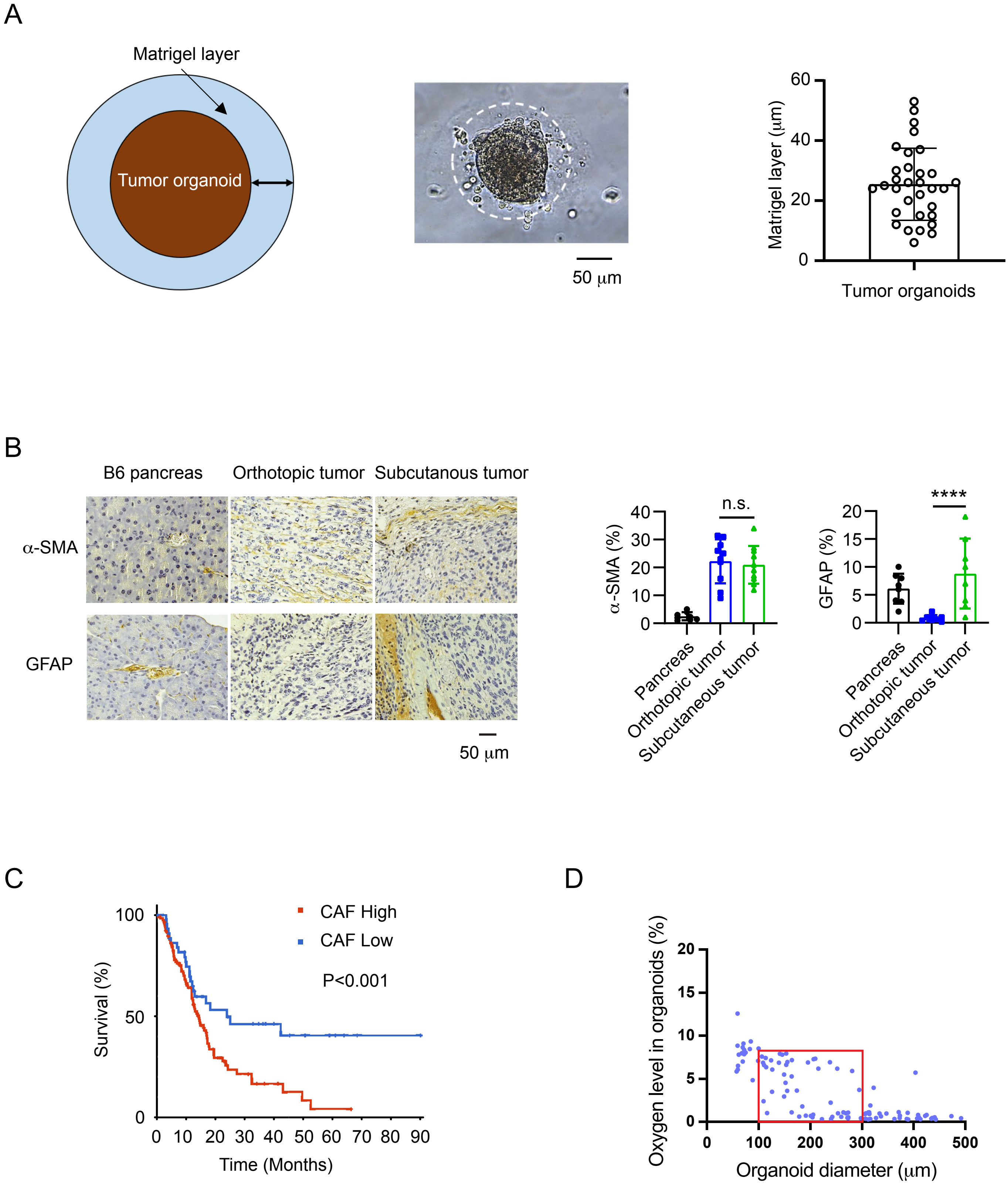

A
Matrigel layer
Tumor organoid
50 μm
60
40
20
0
Matrigel layer (μm)
Tumor organoids
B
B6 pancreas
Orthotopic tumor
Subcutanous tumor
α-SMA
GFAP
50 μm
40
30
20
10
0
α-SMA (%)
n.s.
Pancreas
Orthotopic tumor
Subcutaneous tumor
20
15
10
5
0
GFAP (%)
****
Pancreas
Orthotopic tumor
Subcutaneous tumor
C
100
50
0
Survival (%)
0 10 20 30 40 50 60 70 80 90
Time (Months)
CAF High
CAF Low
P<0.001
D
20
15
10
5
0
Oxygen level in organoids (%)
0 100 200 300 400 500
Organoid diameter (μm)

**Figure S1. Characterization of mouse KPC tumors and derived tumor organoids**

(A), T cell-incorporated KPC tumor organoids. Images were captured under a phase-contrast light microscope (Leica DMi1). Graphic illustration of a tumor organoid with a thin layer of Matrigel and quantification of the Matrigel layer thickness is shown.

(B), Immunohistochemistry (IHC) staining of C57BL/6 mouse pancreas, mouse orthotopic and subcutaneous KPC tumors, and histogram analysis of the $\alpha$SMA or GAFP positive cells from the IHC images (sample size per group: n=8). The data were presented as mean ± SD by One-way ANOVA test. ****, $p < 0.0001$; ns, no significance. Scale bar = 50 $\mu$m.

(C), Progression free survival of human PDA with high and low CAFs, plotted from the cBioPortal database (PDA TCGA, Firehose Legacy). Expression levels of CAF genes (ACTA2, S100A4, PDGFRA, PDGFRB) were used to determine the CAF status in PDA tumors.

(D), Oxygen levels in mouse T cell-incorporated KPC tumor organoids with different sizes.

Fig. S2

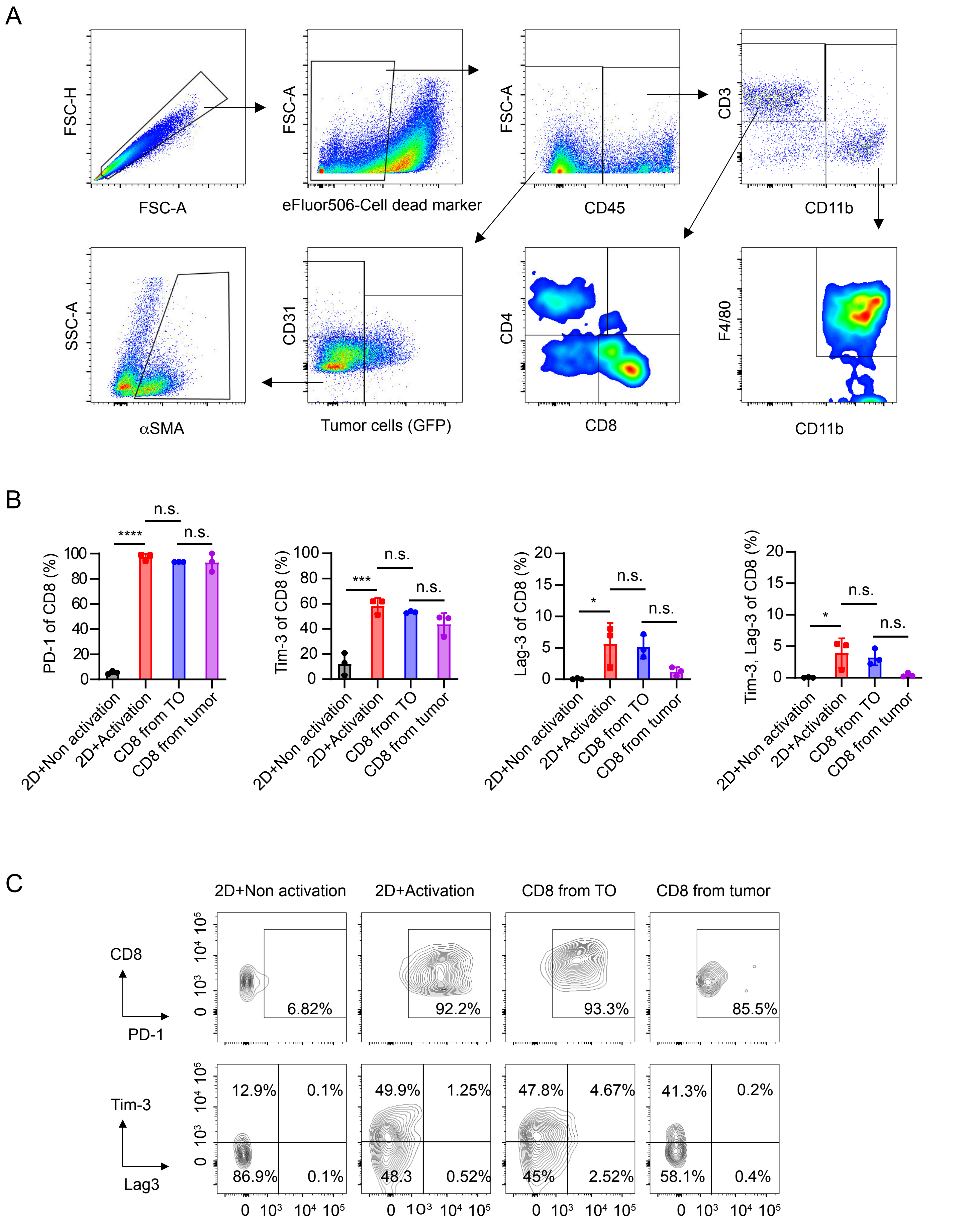
A
FSC-H
FSC-A
FSC-A
eFluor506-Cell dead marker
FSC-A
CD45
CD3
CD11b
SSC-A
αSMA
CD31
Tumor cells (GFP)
CD4
CD8
F4/80
CD11b
B
PD-1 of CD8 (%)
Tim-3 of CD8 (%)
Lag-3 of CD8 (%)
Tim-3, Lag-3 of CD8 (%)
****
***
*
n.s.
2D+Non activation
2D+Activation
CD8 from TO
CD8 from tumor
C
2D+Non activation
2D+Activation
CD8 from TO
CD8 from tumor
CD8
PD-1
6.82%
92.2%
93.3%
85.5%
Tim-3
Lag3
12.9%
0.1%
86.9%
0.1%
49.9%
1.25%
48.3
0.52%
47.8%
4.67%
45%
2.52%
41.3%
0.2%
58.1%
0.4%

**Figure S2. Analysis of T cells incorporated in the KPC tumor organoids**

(A), Flow cytometry gating strategy for cell subtypes in the T cell-incorporated KPC tumor organoids.

(B), Quantification of PD-1, Tim-3 or Lag-3 positive CD8 T cells, isolated from the spleen of OT-I mice or KPC tumor-bearing mice (sample size per group: n=3). Exhaustion (PD-1, Tim-3 or Lag-3) of CD8 T cells from OT-I mouse with or without CD3 and CD28 antibody activation in 2D culture, CD8 T cells from the tumor organoids, and CD8 T cells from the original KPC tumors was measured and analyzed. Data were presented as mean ± SD by One-way ANOVA test. *, $p < 0.05$; ***, $p < 0.001$; ****, $p < 0.0001$; n.s., no significance.

(C), Flow cytometry analysis of the PD-1, Tim-3, or Lag-3 positive CD8 T cells.

Fig. S3

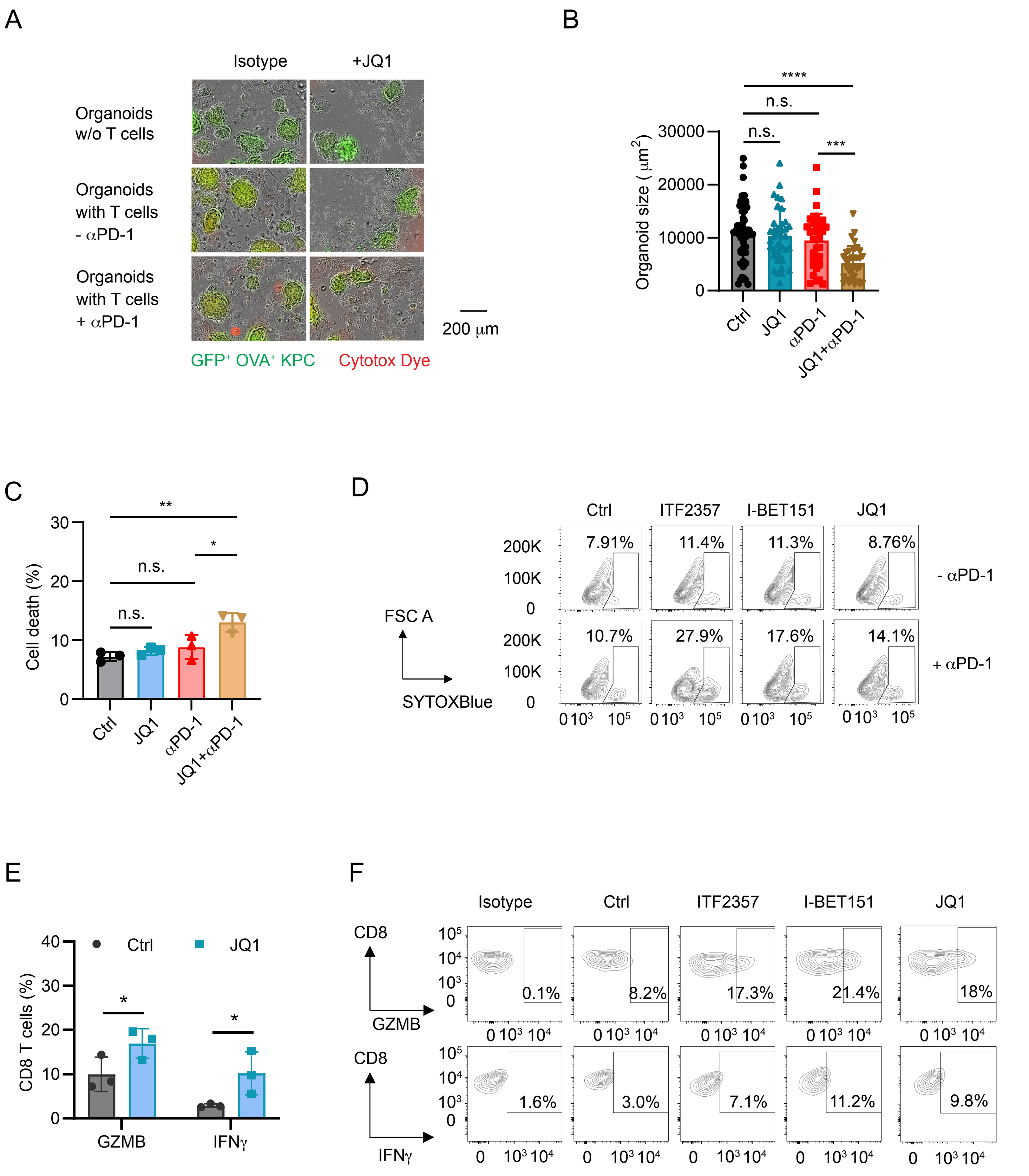

A
Isotype
+JQ1
Organoids w/o T cells
Organoids with T cells - αPD-1
Organoids with T cells + αPD-1
200 μm
GFP+ OVA+ KPC
Cytotox Dye
B
Organoid size (μm2)
****
n.s.
n.s.
***
Ctrl
JQ1
αPD-1
JQ1+αPD-1
C
Cell death (%)
**
*
n.s.
n.s.
Ctrl
JQ1
αPD-1
JQ1+αPD-1
D
Ctrl
ITF2357
I-BET151
JQ1
FSC A
SYTOXBlue
7.91%
11.4%
11.3%
8.76%
- αPD-1
10.7%
27.9%
17.6%
14.1%
+ αPD-1
E
CD8 T cells (%)
Ctrl
JQ1
*
*
GZMB
IFNγ
F
Isotype
Ctrl
ITF2357
I-BET151
JQ1
CD8
GZMB
0.1%
8.2%
17.3%
21.4%
18%
CD8
IFNγ
1.6%
3.0%
7.1%
11.2%
9.8%

**Figure S3. Epigenetic drugs enhance T cell activity in mouse KPC tumor organoids**

(A), Optical and IF images of the $OVA^{+}GFP^{+}KPC$ tumor organoids treated with isotype control, αPD-1, JQ1 or JQ1 + αPD-1, captured by the Incucyte S3 system.

(B), Size quantification of the tumor organoids in (A). Data were presented as mean ± SD by One-way ANOVA test. ***, $p < 0.001$; ****, $p < 0.0001$; n.s., no significance.

(C), The death rate of the $OVA^{+}GFP^{+}KPC$ tumor cells in the T cell-incorporated tumor organoids treated with control, αPD-1, JQ1 or JQ1 + αPD-1 for 48h, measured by flow cytometry (sample size per group: n=3). Data were presented as mean ± SD by One-way ANOVA test. *, $p < 0.05$; **, $p < 0.01$; n.s., no significance.

(D), Representative flow cytometry data showing the $OVA^{+}GFP^{+}$ KPC cell death rate in the T cell-incorporated tumor organoids.

(E), GZMB and IFNγ positive CD8 T cells in the tumor organoids treated with control or JQ1 (sample size per group: n=3). Data were presented as mean ± SD by Two-way ANOVA test. *, $p < 0.05$.

(F), Representative flow cytometry data showing the GZMB and IFNγ positive CD8 T cells in the tumor organoids.

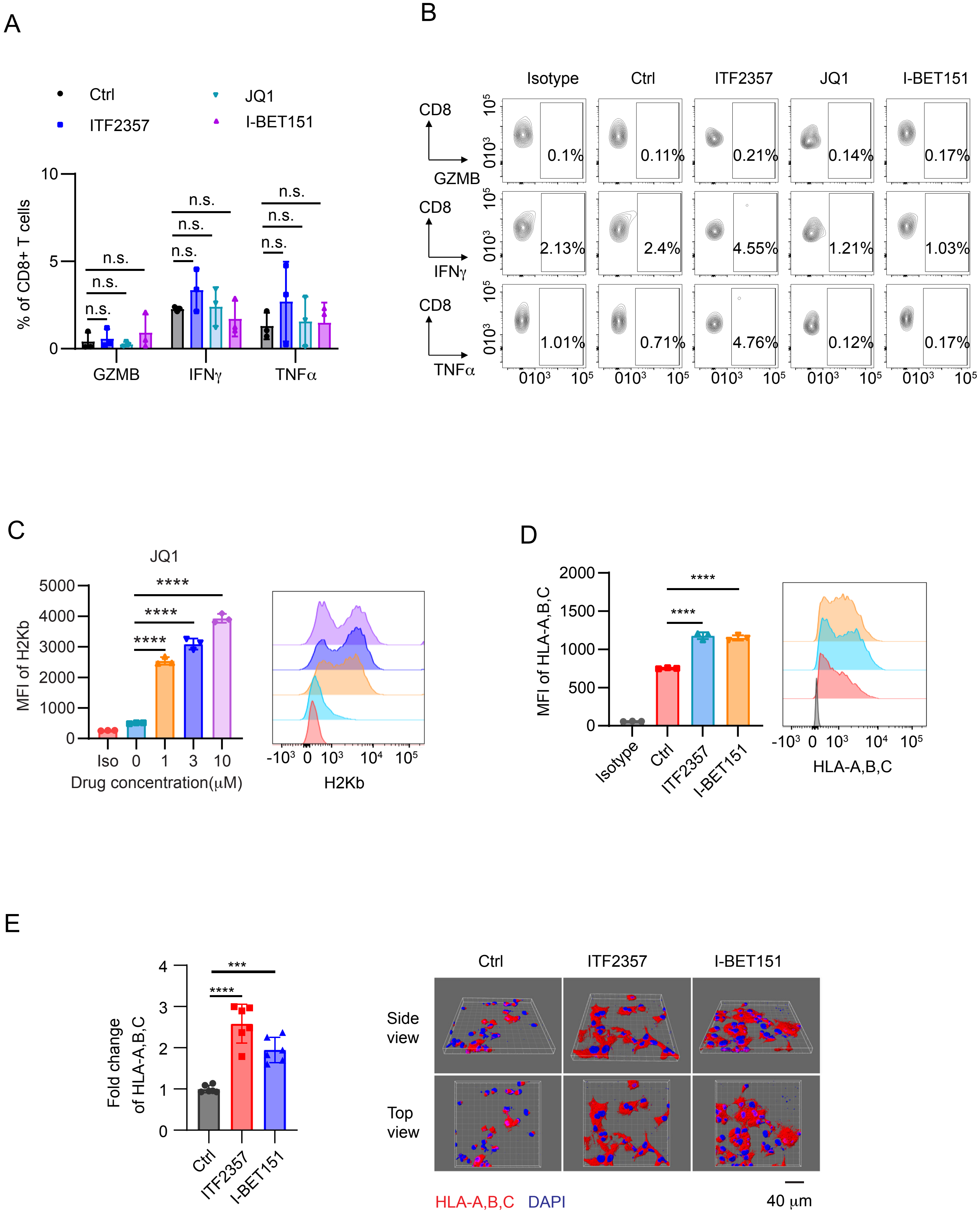
A
Ctrl
ITF2357
JQ1
I-BET151
% of CD8+ T cells
n.s.
GZMB
IFNγ
TNFα
B
Isotype
Ctrl
ITF2357
JQ1
I-BET151
CD8
GZMB
IFNγ
TNFα
0.1%
0.11%
0.21%
0.14%
0.17%
2.13%
2.4%
4.55%
1.21%
1.03%
1.01%
0.71%
4.76%
0.12%
0.17%
C
JQ1
MFI of H2Kb
****
Iso
Drug concentration(μM)
H2Kb
D
MFI of HLA-A,B,C
****
Isotype
Ctrl
ITF2357
I-BET151
HLA-A,B,C
E
Fold change of HLA-A,B,C
***
****
Ctrl
ITF2357
I-BET151
Side view
Top view
HLA-A,B,C
DAPI
40 μm

**Figure S4. The treatment effects of epigenetic drugs on mouse T cells**

(A), GZMB, IFNγ and TNFα expression levels of the control, ITF2357, I-BET151 or JQ1 treated CD8 T cells that were isolated from the spleen of OT-I mouse (sample size per group: n=3). Data were presented as mean ± SD by Two-way ANOVA test. n.s., no significance.

(B), Representative flow cytometry data of (A).

(C), The mean fluorescence intensity (MFI) of H-2Kb on the mouse $OVA^{+}GFP^{+}$ KPC cells treated with control or JQ1 and flow cytometry analysis (sample size per group: n=3). Data were presented as mean ± SD by One-way ANOVA test. ****, $p < 0.0001$.

(D), The mean fluorescence intensity (MFI) of HLA-A,B,C on the human PANC-1 cells treated with control, ITF2357, or I-BET151 (sample size per group: n=3). Data were presented as mean ± SD by One-way ANOVA test. ****, $p < 0.0001$.

(E), The relative expression levels of HLA-A,B,C on the human PANC-1 cells treated with control, ITF2357, or I-BET151, analyzed with images captured by confocal microscope (sample size per group: n=6). Data were presented as mean ± SD by One-way ANOVA test. ***, $p < 0.001$; ****, $p < 0.0001$.

Fig. S5

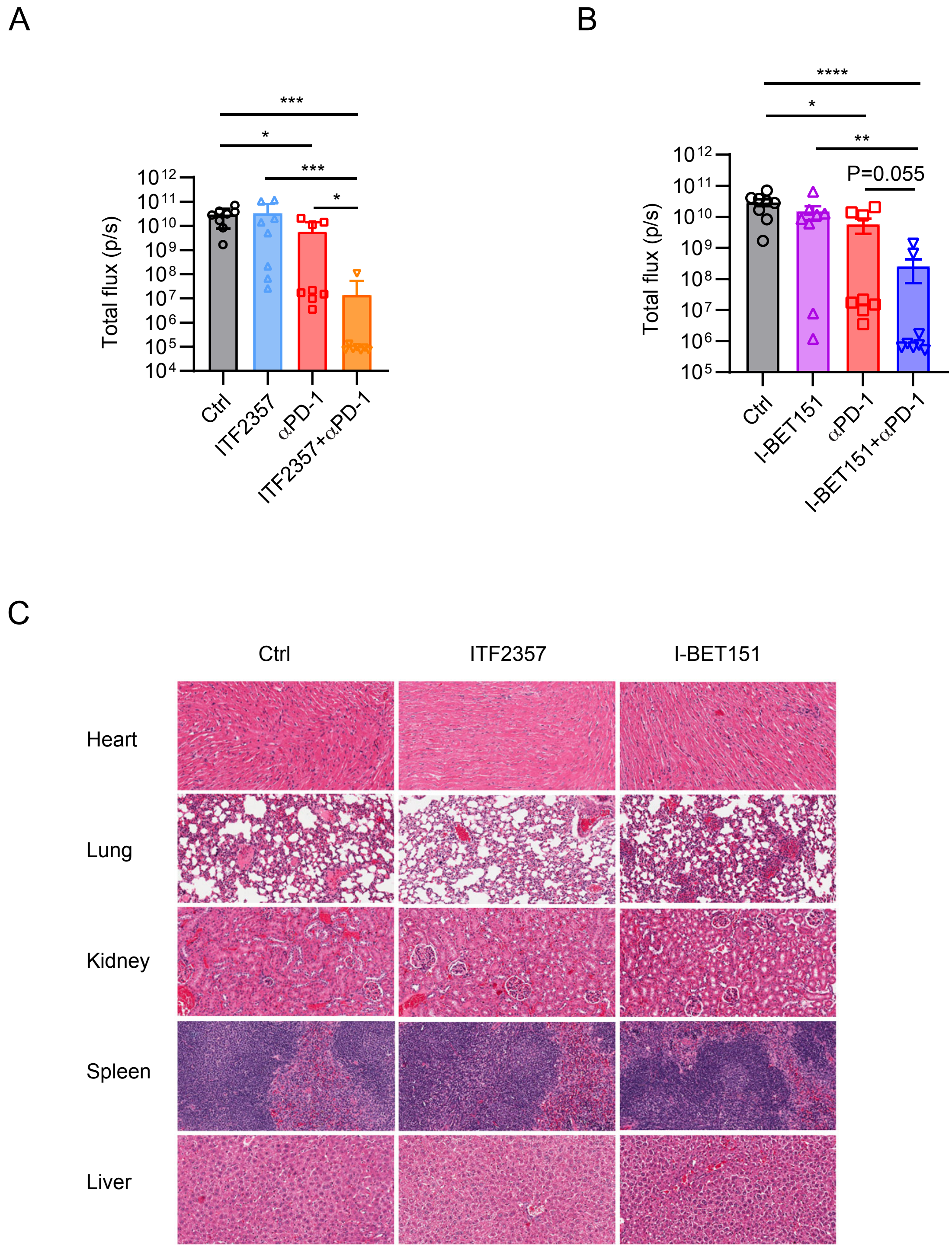
A
B
C
Total flux (p/s)
***
*
***
*
Ctrl
ITF2357
αPD-1
ITF2357+αPD-1
****
*
**
P=0.055
I-BET151
I-BET151+αPD-1
Ctrl
ITF2357
I-BET151
Heart
Lung
Kidney
Spleen
Liver

**Figure S5. ITF2357 or I-BET151 augments anti-tumor activity of immune checkpoint blockade in KPC tumor-bearing mice**

(A), The total flux of KPC tumors from C57BL/6 mice treated with isotype control, αPD-1, ITF2357 or ITF2357 + αPD-1, measured at day 43 post orthotopic injection of KPC cells into C57BL/6 mice (sample size per group: n=8). Data were presented as mean ± SD by One-way ANOVA test. *, $p < 0.05$; ***, $p < 0.001$.

(B), The total flux of KPC tumors in C57BL/6 mice treated with isotype control, αPD-1, I-BET151 or I-BET151 + αPD-1, measured at day 43 (sample size per group: n=8). Data were presented as mean ± SD by One-way ANOVA test. *, $p < 0.05$; **, $p < 0.01$; ****, $p < 0.0001$.

(C), Histological evaluation on the indicated organs of C57BL/6 mice treated with control, ITF2357 or I-BET151.

Fig. S6

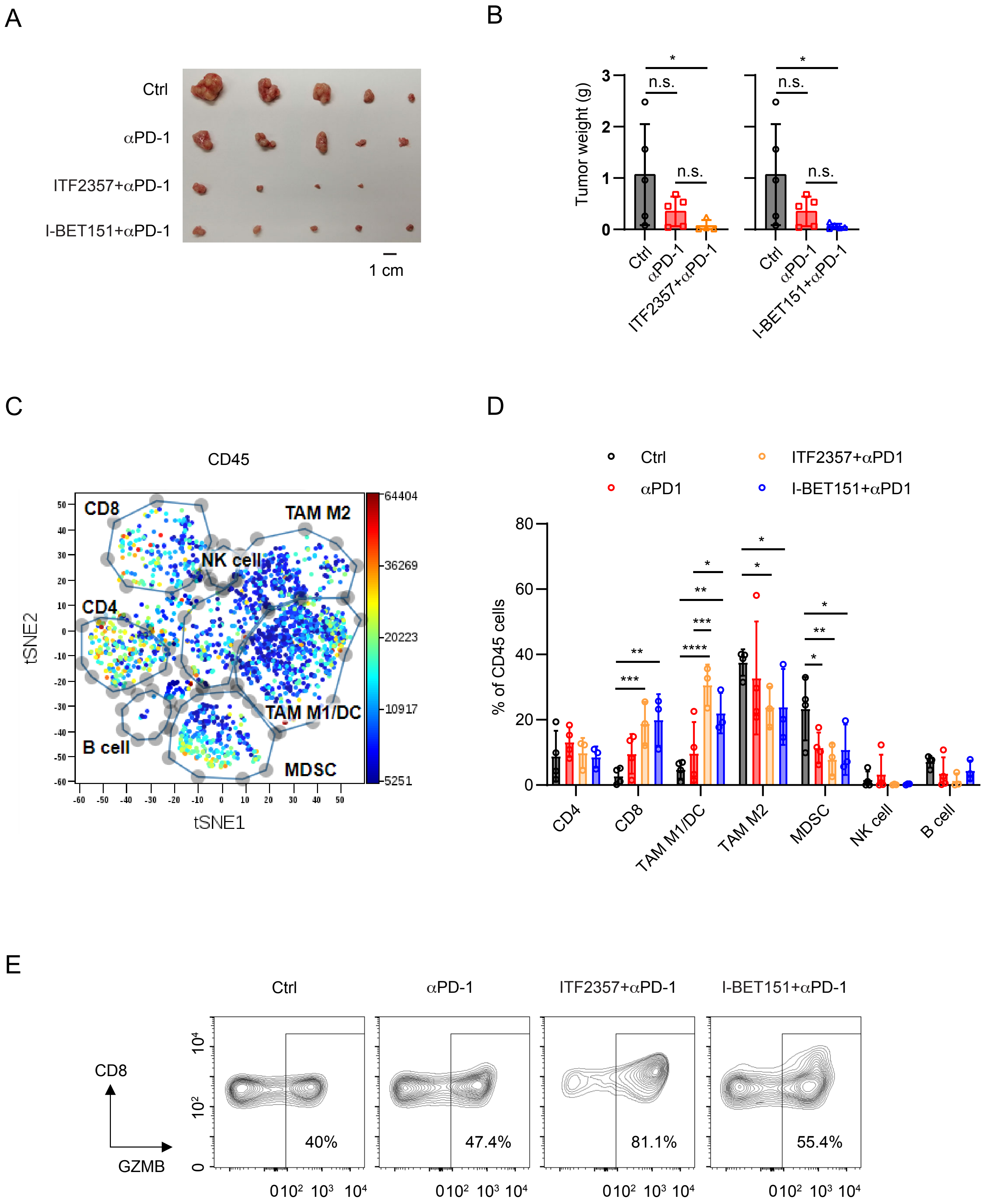
A
Ctrl
αPD-1
ITF2357+αPD-1
I-BET151+αPD-1
1 cm
B
Tumor weight (g)
n.s.
*
Ctrl
αPD-1
ITF2357+αPD-1
I-BET151+αPD-1
C
CD45
CD8
TAM M2
NK cell
CD4
B cell
TAM M1/DC
MDSC
tSNE1
tSNE2
64404
36269
20223
10917
5251
D
Ctrl
αPD1
ITF2357+αPD1
I-BET151+αPD1
% of CD45 cells
CD4
CD8
TAM M1/DC
TAM M2
MDSC
NK cell
B cell
E
Ctrl
αPD-1
ITF2357+αPD-1
I-BET151+αPD-1
CD8
GZMB
40%
47.4%
81.1%
55.4%

**Figure S6. Immune profiling analysis of orthotopic KPC tumors**

(A and B), Gross tumor images (A) and tumor weight (B) of the KPC tumors harvested from the C57BL/6 mice treated with control, αPD-1, ITF2357 + αPD-1 or I-BET151 + αPD-1 (sample size per group: n=5). The tumors were collected on day 30 post-orthotopic injection. Data shown as mean ± SD by One-way ANOVA test. *, $p < 0.05$; n.s., no significance.

(C), Gating strategy for immune cells in the KPC tumors harvested from the tumor-bearing C57BL/6 mice. The data were analyzed using t-SNE in the Cytobank platform.

(D), The immune cell subtype populations in the KPC tumors treated with isotype antibody control, αPD-1, ITF2357 + αPD-1 or I-BET151 + αPD-1, determined by t-SNE analysis (sample size per group: n=3 or 4). Data were analyzed using two-way ANOVA and shown as mean ± SD. *, p <0.05; **, p <0.01; ***, p <0.001; ****, p <0.0001.

(E), The flow cytometry analysis of the GZMB expression levels on CD8 T cells in the KPC tumors.

Fig. S7

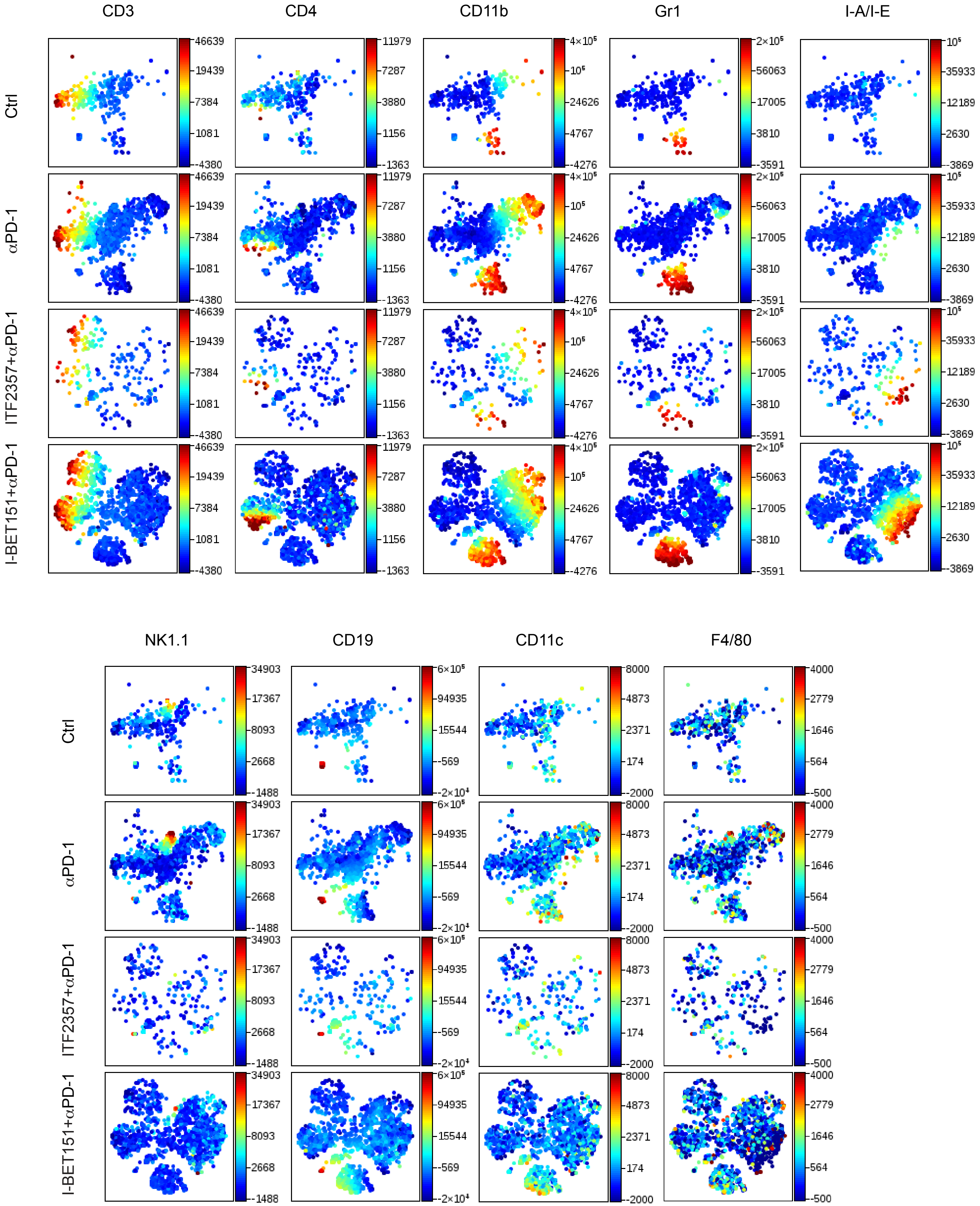
CD3
CD4
CD11b
Gr1
I-A/I-E
Ctrl
αPD-1
ITF2357+αPD-1
I-BET151+αPD-1
46639
19439
7384
1081
-4380
11979
7287
3880
1156
-1363
4×10⁵
10⁵
24626
4767
-4276
2×10⁵
56063
17005
3810
-3591
10⁵
35933
12189
2630
-3869
NK1.1
CD19
CD11c
F4/80
34903
17367
8093
2668
-1488
6×10⁵
94935
15544
-569
-2×10⁴
8000
4873
2371
174
-2000
4000
2779
1646
564
-500

**Figure S7. t-SNE representation of immune cell subtypes**

Fig. S8

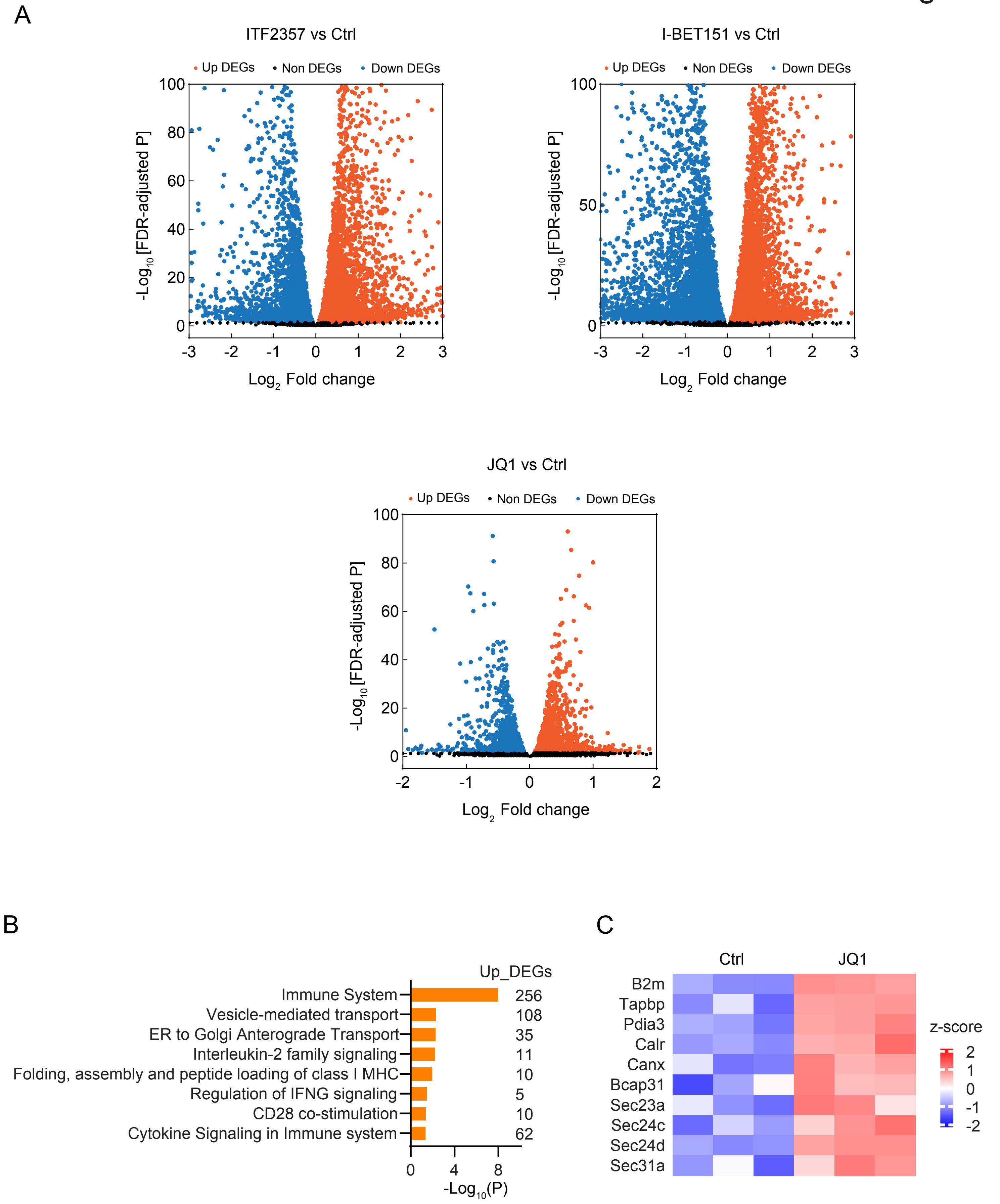
A
ITF2357 vs Ctrl
Up DEGs
Non DEGs
Down DEGs
-Log10 [FDR-adjusted P]
Log2 Fold change
I-BET151 vs Ctrl
Up DEGs
Non DEGs
Down DEGs
-Log10 [FDR-adjusted P]
Log2 Fold change
JQ1 vs Ctrl
Up DEGs
Non DEGs
Down DEGs
-Log10 [FDR-adjusted P]
Log2 Fold change
B
Up_DEGs
Immune System 256
Vesicle-mediated transport 108
ER to Golgi Anterograde Transport 35
Interleukin-2 family signaling 11
Folding, assembly and peptide loading of class I MHC 10
Regulation of IFNG signaling 5
CD28 co-stimulation 10
Cytokine Signaling in Immune system 62
-Log10(P)
C
Ctrl
JQ1
B2m
Tapbp
Pdia3
Calr
Canx
Bcap31
Sec23a
Sec24c
Sec24d
Sec31a
z-score

**Figure S8. RNA-seq data analysis of mouse KPC cells treated with epigenetic drugs**

(A), Volcano plots of RNA-seq data showing the DEGs from the ITF2357, I-BET151 or JQ1 treated group in comparison with the control group.

(B), Analysis of up-regulated DEGs pathways of the control and JQ1 treatment groups from the RNA-seq data.

(C), Heatmap of the DEGs pathway of Folding, Assembly and Peptide loading of class I MHC-I of the control and JQ1 treatment groups.

Fig. S9

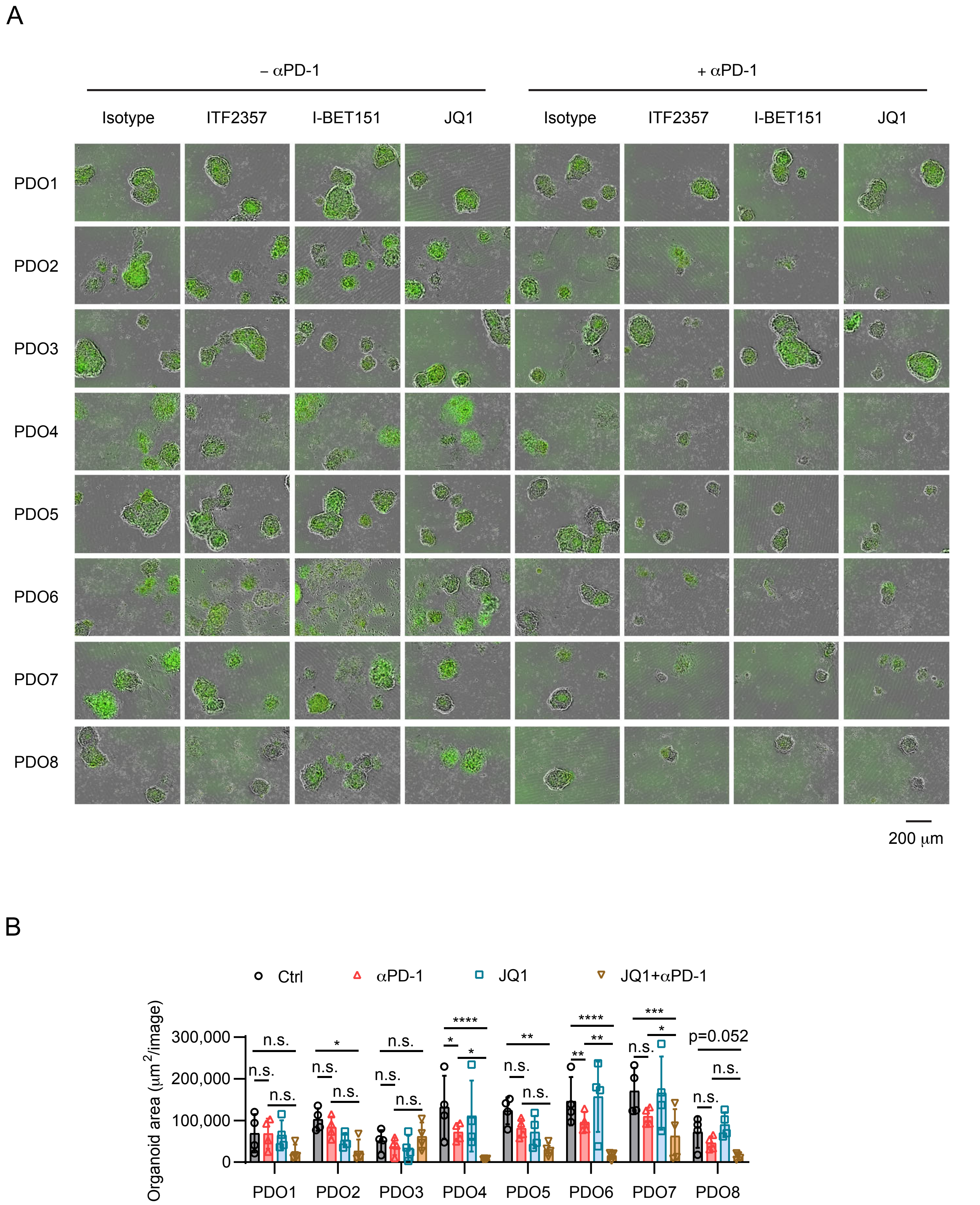

A
– αPD-1
+ αPD-1
Isotype
ITF2357
I-BET151
JQ1
Isotype
ITF2357
I-BET151
JQ1
PDO1
PDO2
PDO3
PDO4
PDO5
PDO6
PDO7
PDO8
200 μm
B
Ctrl
αPD-1
JQ1
JQ1+αPD-1
Organoid area (μm2/image)
300,000
200,000
100,000
0
n.s.
*
****
**
***
p=0.052
PDO1
PDO2
PDO3
PDO4
PDO5
PDO6
PDO7
PDO8

**Figure S9. Epigenetic drugs enhance the cytotoxicity of autologous T cells incorporated in the PDOs**

(A), Optical and fluorescence images of T cell-incorporated PDOs, treated with isotype control, αPD-1, ITF2357, I-BET151, JQ1, ITF2357 + αPD-1, I-BET151 + αPD-1 or JQ1 + αPD-1 for 48h, captured by the Incucyte S3 system. The Cytolight Green dye stains live cells.

(B), Quantification of T cell-mediated cytotoxicity in the PDOs treated with control, JQ1, αPD-1 or JQ1 + αPD-1. Smaller areas of PDOs indicate higher cytotoxicity (sample size per group: n=4). Note that the data of control and αPD-1 groups are shared with those in Figure 8C from the same set of experiments. Data were presented as mean ± SD by Two-way ANOVA test. *, $p < 0.05$; **, $p < 0.01$; ***, $p < 0.001$; ****, $p < 0.0001$; ns, no significance.

**Table S1. Epigenetic Compounds (Cayman Chemical)**

Item No.11076; Batch No. 0522205

| Number | Drug Name | Catalog# |
|---|---|---|
| 1 | (+)-Abscisic Acid | 10073 |
| 2 | 3-amino Benzamide | 10397 |
| 3 | SB939 | 10443 |
| 4 | PCI 34051 | 10444 |
| 5 | 4-iodo-SAHA | 10495 |
| 6 | Sirtinol | 10523 |
| 7 | C646 | 10549 |
| 8 | Tubastatin A (trifluoroacetate salt) | 10559 |
| 9 | Garcinol | 10566 |
| 10 | Ellagic Acid | 10569 |
| 11 | Scriptaid | 10572 |
| 12 | Apicidin | 10575 |
| 13 | HC Toxin | 10576 |
| 14 | UNC0321 (trifluoroacetate salt) | 10582 |
| 15 | (−)-Neplanocin A | 10584 |
| 16 | Cl-Amidine (trifluoroacetate salt) | 10599 |
| 17 | F-Amidine (trifluoroacetate salt) | 10610 |
| 18 | JGB1741 | 10641 |
| 19 | coumarin-SAHA | 10671 |
| 20 | I-BET762 | 10676 |
| 21 | UNC0638 | 10734 |
| 22 | Phthalazinone pyrazole | 10735 |
| 23 | Isoliquiritigenin | 10739 |
| 24 | CCG-100602 | 10787 |
| 25 | CAY10669 | 10974 |
| 26 | Zebularine | 10975 |
| 27 | Delphinidin (chloride) | 11012 |
| 28 | ITF 2357 | 11045 |
| 29 | UNC0631 | 11084 |
| 30 | UNC0646 | 11085 |
| 31 | Methylstat (hydrate) | 11091 |
| 32 | 3-Deazaneplanocin A (hydrochloride) | 11102 |
| 33 | Suramin (sodium salt) | 11126 |
| 34 | Nicotinamide | 11127 |
| 35 | 2,4-Pyridinedicarboxylic Acid | 11138 |
| 36 | PFI-1 | 11155 |
| 37 | 5-Azacytidine | 11164 |
| 38 | SGI-1027 | 11165 |
| 39 | Decitabine | 11166 |
| 40 | I-BET151 | 11181 |

| | | |
|---|---|---|
| 41 | (+)-JQ1 | 11187 |
| 42 | (−)-JQ1 | 11232 |
| 43 | BSI-201 | 11304 |
| 44 | 1-Naphthoic Acid | 11322 |
| 45 | Sodium 4-Phenylbutyrate | 11323 |
| 46 | Rucaparib (phosphate) | 11570 |
| 47 | IOX1 | 11572 |
| 48 | MI-2 (hydrochloride) | 11620 |
| 49 | MI-nc (hydrochloride) | 11621 |
| 50 | Gemcitabine | 11690 |
| 51 | Lomeguatrib | 11732 |
| 52 | GSK4112 | 11931 |
| 53 | Octyl-α-ketoglutarate | 11970 |
| 54 | Daminozide | 12033 |
| 55 | GSK-J1 (sodium salt) | 12054 |
| 56 | GSK-J2 (sodium salt) | 12056 |
| 57 | GSK-J4 (hydrochloride) | 12073 |
| 58 | CI-994 | 12084 |
| 59 | CPTH2 (hydrochloride) | 12086 |
| 60 | Etoposide | 12092 |
| 61 | Lestaurtinib | 12094 |
| 62 | Butyrolactone 3 | 12095 |
| 63 | Valproic Acid (sodium salt) | 13033 |
| 64 | Tenovin-1 | 13085 |
| 65 | Tenovin-6 | 13086 |
| 66 | Sodium Butyrate | 13121 |
| 67 | BIX01294 (hydrochloride hydrate) | 13124 |
| 68 | Anacardic Acid | 13144 |
| 69 | AGK2 | 13145 |
| 70 | CAY10603 | 13146 |
| 71 | Splitomicin | 13168 |
| 72 | CBHA | 13172 |
| 73 | M 344 | 13174 |
| 74 | Oxamflatin | 13176 |
| 75 | Salermide | 13178 |
| 76 | Mirin | 13208 |
| 77 | Pimelic Diphenylamide 106 | 13212 |
| 78 | KD 5170 | 13214 |
| 79 | Panobinostat | 13280 |
| 80 | MS-275 | 13284 |
| 81 | HNHA | 13295 |
| 82 | RG-108 | 13302 |
| 83 | 2’,3’,5’-triacetyl-5-Azacytidine | 13373 |
| 84 | S-Adenosylhomocysteine | 13603 |

| | | |
|---|---|---|
| 85 | UNC0224 | 13631 |
| 86 | Chidamide | 13686 |
| 87 | Tubacin | 13691 |
| 88 | 3-Deazaneplanocin A | 13828 |
| 89 | Sinefungin | 13829 |
| 90 | Pyroxamide | 13870 |
| 91 | N-Oxalylglycine | 13944 |
| 92 | WDR5-0103 | 13945 |
| 93 | EPZ005687 | 13966 |
| 94 | SGC0946 | 13967 |
| 95 | UNC1215 | 13968 |
| 96 | AK-7 | 14004 |
| 97 | GSK343 | 14094 |
| 98 | Bromosporine | 14119 |
| 99 | GSK2801 | 14120 |
| 100 | SIRT1/2 Inhibitor IV | 14407 |
| 101 | I-CBP112 (hydrochloride) | 14468 |
| 102 | SGC-CBP30 | 14469 |
| 103 | UNC0642 | 14604 |
| 104 | UNC1999 | 14621 |
| 105 | I-PFI-2 (hydrochloride) | 14678 |
| 106 | HPOB | 15066 |
| 107 | 2-hexyl-4-Pentynoic Acid | 15205 |
| 108 | PFI-3 | 15267 |
| 109 | JIB-04 | 15338 |
| 110 | CAY10683 | 15403 |
| 111 | GSK126 | 15415 |
| 112 | CPI-203 | 15479 |
| 113 | 6-Thioguanine | 15774 |
| 114 | Tubastatin A | 15785 |
| 115 | 3,3'-Diindolylmethane | 15927 |
| 116 | OTX015 | 15947 |
| 117 | 5-Methylcytidine | 16111 |
| 118 | AGK7 | 16152 |
| 119 | 5-Methyl-2'-deoxycytidine | 16166 |
| 120 | EPZ5676 | 16175 |
| 121 | MC 1568 | 16265 |
| 122 | α-Hydroxyglutaric Acid (sodium salt) | 16374 |
| 123 | S-(5'-Adenosyl)-L-methionine (tosylate) | 16376 |
| 124 | RVX-208 | 16424 |
| 125 | CUDC-101 | 16426 |
| 126 | LAQ824 | 16427 |
| 127 | Nullscript | 16433 |
| 128 | GSK-LSD1 (hydrochloride) | 16439 |

| | | |
|---|---|---|
| 129 | RGFP966 | 16917 |
| 130 | BRD73954 | 16919 |
| 131 | *trans*-Resveratrol | 70675 |
| 132 | DMOG | 71210 |
| 133 | Trichostatin A | 89730 |
| 134 | CAY10398 | 89740 |
| 135 | RSC-133 | 9001839 |
| 136 | BML-210 | 10005019 |
| 137 | Piceatannol | 10009366 |
| 138 | CAY10591 | 10009797 |
| 139 | EX-527 | 10009798 |
| 140 | SAHA | 10009929 |
| 141 | 2-PCPA (hydrochloride) | 10010494 |

**Table S2. The antibody panel for Cytek Aurora analysis**

| Number | Channel | Antibody name | Dye | Company | Catalog# |
|---|---|---|---|---|---|
| 1 | V1 | PD-1 | BV421 | BioLegend | 135218 |
| 2 | V2 | CD25 | Superbright436 | Invitrogen | 2470828 |
| 3 | V3 | CD11b | eFluor 450 | Invitrogen | 2403283 |
| 4 | V4 | EpCAM | BV480 | BD Biosciences | 746367 |
| 5 | V5 | Viable | eFluor 506 | ThermoFisher | 65-0866-14 |
| 6 | V7 | CD69 | BV510 | BioLegend | 104532 |
| 7 | V8 | CD11c | BV570 | BioLegend | 117331 |
| 8 | V10 | Gr1 | BV605 | BioLegend | 108440 |
| 9 | V11 | Ly108 | BV650 | BioLegend | 740628 |
| 10 | V13 | CD3 | BV711 | BioLegend | 100241 |
| 11 | V14 | CD103 | BV750 | BioLegend | 747478 |
| 12 | V15 | CD62L | BV785 | BioLegend | 104440 |
| 13 | B1 | CD19 | BB515 | BioLegend | 564509 |
| 14 | B2 | $GFP^+$ KPC cell | GFP | | |
| 15 | B3 | CD45 | AF532 | Invitrogen | 58-0451-82 |
| 16 | B8 | IAIE | PerCP | BioLegend | 107624 |
| 17 | B9 | F4/80 | PerCPCy5.5 | BioLegend | 123128 |
| 18 | B10 | GZMB | PerCP-eFluor™ 710 | Invitrogen | 46-8898-80 |
| 19 | YG1 | TNFα | PE | BioLegend | 506306 |
| 20 | YG3 | CD8 | PE-eFluor 610 | Invitrogen | 61-0081-80 |
| 21 | YG5 | CD44 | PE/Cy5 | BioLegend | 103010 |
| 22 | YG9 | CD31 | PE/Cy7 | Invitrogen | 25-0311-81 |
| 23 | R1 | IFNγ | APC | BioLegend | 505810 |
| 24 | R2 | α-SMA | AF647 | Cell Signaling Technology | 76113S |
| 25 | R4 | NK1.1 | AF700 | BioLegend | 108730 |
| 26 | R7 | CD4 | APC/CY7 | BioLegend | 100414 |

**Table S3. Clinical information of human pancreatic cancer samples.**

| Sample | Sex | Surgical information | Diagnosis |
|---|---|---|---|
| 1 | M | Whipple | Pancreatic adenocarcinoma |
| 2 | M | Pancreatectomy | Anaplastic undifferentiated carcinoma |
| 3 | F | Whipple | Pancreatic adenocarcinoma |
| 4 | F | Whipple | Pancreatic adenocarcinoma |
| 5 | M | Whipple | Pancreatic adenocarcinoma |
| 6 | F | Whipple | Pancreatic adenocarcinoma |
| 7 | F | Distal pancreatectomy and splenectomy | Pancreatic adenocarcinoma |
| 8 | F | Pancreaticoduodenectomy | Pancreatic adenocarcinoma |

**Table S4. Components in the culture medium for mouse pancreatic tumor organoids**

| Products | Name | Company | Catalog | Concentration |
|---|---|---|---|---|
| 1 | GlutaMAX Supplement | ThermoFisher | 35050061 | 1x |
| 2 | B-27™ Supplement (50X) | Sigma | 17504044 | 1x |
| 3 | N-acetyl-L-cysteine | Sigma | A7250 | 1 mM |
| 4 | Recombinant murine EGF | PeproTech | 315-09 | 5 μg/ml |
| 5 | Wnt-3A, R-spondin 3, and noggin conditioned medium | ATCC | CRL-3276 | 10% |
| 6 | FGF10 | BioLegend | 751004 | 10 ng/ml |
| 7 | Recombinant murine FGF-10 | Sigma | PMG0034 | 5 ng/ml |
| 8 | A83-01 | Tocris | 2939 | 500 nM |
| 9 | Nicotinamide | Sigma | N3376 | 10 mM |

**Table S5. Key resources**

| Reagent or resource | Source | Identifier |
|---|---|---|
| **Antibodies** | | |
| Recombinant-EPCAM antibody | Abcam | Cat# ab32392 |
| AF647-conjugated Donkey anti-rabbit IgG | BioLegend | Cat# 406414 |
| AF 647-conjugated anti-Cytokeratin 19 | BioLegend | Cat# 628506 |
| Spark YG 570-conjugated anti-mouse CD31 | BioLegend | Cat# 102531 |
| PE-conjugated anti-human CD31 | BioLegend | Cat# 303106 |
| AF488 -conjugated anti-human CD68 | BioLegend | Cat# 333811 |
| Spark YG 570-conjugated anti-human CD8a | BioLegend | Cat# 372910 |
| Spark YG 570-conjugated anti-mouse F4/80 | BioLegend | Cat# 123159 |
| AF488-conjugated anti-mouse CD8a | BioLegend | Cat# 100723 |
| FITC-conjugated Mouse α-Smooth Muscle Actin | GeneTex | Cat# GTX72531 |
| AF647-conjugated anti-mouse CD8a | BioLegend | Cat# 100724 |
| AF647-conjugated anti-mouse CD4 | BioLegend | Cat# 100533 |
| anti-mouse CD8α antibody, rabbit monoclonal | Cell Signaling Technology | Cat# 98941S; Clone: D4W2Z |
| CD4 Rabbit mAb | Cell Signaling Technology | Cat# 25229; Clone: D7D2Z |
| α-Smooth Muscle Actin XP Rabbit mAb | Cell Signaling Technology | Cat# 19245S; Clone: D4K9N |
| GFAP Mouse mAb | Cell Signaling Technology | Cat# 3670S; Clone:GA5 |
| AF647-conjugated anti-mouse CD326 | BioLegend | Cat# 118212 |
| AF647-conjugated α-Smooth Muscle Actin XP Rabbit mAb | Cell Signaling Technology | Cat# 76113S; Clone: D4K9N |
| AF594-conjugated anti-mouse CD31 | BioLegend | Cat# 102520 |
| BV605-conjugated anti-mouse CD45 | BioLegend | Cat# 103139 |
| PE/Cy7-conjugated anti-mouse CD3 | BioLegend | Cat# 100220 |
| AF700-conjugated anti-mouse CD4 | BioLegend | Cat# 100430 |
| APC/Cy7-conjugated anti-mouse CD8 | BioLegend | Cat# 100714 |
| PE-conjugated anti-mouse/human CD11b | BioLegend | Cat# 101208 |
| AF647-conjugated anti-mouse CD11c | BioLegend | Cat# 117312 |

| | | |
|---|---|---|
| PerCP/Cy5.5-conjugated anti-mouse F4/80 | BioLegend | Cat# 123128 |
| BV421-conjugated anti-mouse I-A/I-E | BioLegend | Cat# 107631 |
| BV650-conjugated anti-mouse CD19 | BioLegend | Cat# 115541 |
| BV605-conjugated anti-mouse Gr1 | BioLegend | Cat# 108440 |
| AF700-conjugated anti-mouse NK1.1 | BioLegend | Cat# 108730 |
| APC-conjugated anti-mouse IFNγ | BioLegend | Cat# 505810 |
| PE-conjugated anti-mouse TNFα | BioLegend | Cat# 506306 |
| PerCP/Cyanine5.5-conjugated anti-human/mouse Granzyme B | BioLegend | Cat# 372212 |
| AF647-conjugated anti-mouse H-2Kb | BioLegend | Cat# 116512 |
| APC-conjugated anti-mouse H-2Kb bound to SIINFEKL antibody | BioLegend | Cat# 141606 |
| PE/Cyanine7-conjugated anti-human CD45 | BioLegend | Cat# 368532 |
| AF647-conjugated anti-human CD3 | BioLegend | Cat# 300422 |
| AF700-conjugated anti-human CD4 | BioLegend | Cat# 357418 |
| APC/Cy7-conjugated anti-human CD8a | BioLegend | Cat# 300926 |
| AF647-conjugated Anti-Human HLA-A,B,C | BioLegend | Cat# 311414 |
| anti-mouse PD-1 | Bioxcell | Cat# BE0146;<br>Clone: RMP1-14 |
| Rat IgG2a isotype control | Bioxcell | Cat# BE0089 |
| Beta-2 microglobulin antibody | Abcam | Cat# Ab175031 |
| Beta-Actin antibody | Abgent | Cat# AM1829b |
| **Chemicals, Peptides, and Proteins** | | |
| Mouse IL-2 | BioLegend | Cat# 575402 |
| Human IL-2 | Peprotech | Cat# 200-02 |
| penicillin/streptomycin solution | HyClone | Cat# SV30010 |
| Matrigel | Corning | Cat# 356255 |
| Image-iT$^{TM}$ Red Hypoxia reagent | Invitrogen | Cat# H10498 |
| phorbol 12-myristate 13-acetate | Sigma | Cat# 16561-29-8 |
| Ionomycin | Sigma | Cat# 56092-82-1 |
| Brefeldin A | BioLegend | Cat# 420601 |
| Lipofectamine 3000 reagent | ThermoFisher Scientific | Cat# 25530049 |
| TRIzol | Ambion | Cat# 15596018 |

| | | |
|---|---|---|
| Citrate buffer antigen retriever | Sigma | Cat# C9999-1000ml |
| Tween 20 | ThermoFisher Scientific | Cat# BP337-500 |
| eBioscience™ Fixable Viability Dye eFluor™ 506 | ThermoFisher Scientific | Cat# 65-0866-14 |
| Incucyte Cytotox Red Dye | Sartorius | Cat# 4632 |
| Incucyte Cytolight Rapid Dye (Green) | Sartorius | Cat# 4705 |
| SYTOX$^{TM}$ Blue dead cells | Invitrogen | Cat# 2078615 |
| DAPI (4',6-Diamidino-2-Phenylindole, Dihydrochloride) | ThermoFisher Scientific | Cat# D1306 |
| **Commercial assays and Consumables** | | |
| Mouse tumor dissociation kit | Milenyi Biotec | Cat# 130-096-730 |
| Human tumor dissociation kit | Milenyi Biotec | Cat# 130-095-929 |
| Pan T cell isolation kit II (mouse) | Milenyi Biotec | Cat# 130-095-130 |
| Pan T cell isolation kit II (human) | Milenyi Biotec | Cat# 130-096-535 |
| RNeasy mini-isolation kit | QIAGEN | Cat# 157029493 |
| QIAseq FastSelect rRNA Removal HMR Kit | QIAGEN | Cat# 334387 |
| KAPA RNA Hyper Prep Kit | Roche Corporate | Cat# KK8541 |
| QscriptXLT cDNA super Mix | Quantabio | Cat# 66141329 |
| SYBR Green fastmix low Rox | TaKaRa | Cat# 4385610 |
| Cytofix/cytoperm fixation/permeabilization kit | BD Biosciences | Cat# 554714 |
| DAB substrate Kit Peroxidase | Vector | Cat# SK-4100 |
| Epigenetic drug library | Cayman Chemical | Cat# 11071 |
| Human CD8 magnetic beads | Milenyi Biotec | Cat# 130-045-201 |
| Mouse CD8 magnetic beads | Milenyi Biotec | Cat# 130-117-044 |
| BD comp Beads Negative Control | BD Biosciences | Cat# 51-90-9001291 |
| BD comp Beads Anti-Mouse Ig,κ | BD Biosciences | Cat# 51-90-9001229 |
| BD comp Beads Anti-Rat and Anti-Hamster Ig,κ | BD Biosciences | Cat# 51-90-9000949 |
| Picro-Sirius Red Solution | Abcam | Cat# ab246832 |
| Prolong Gold anti-fade medium | Invitrogen | Cat# P36930 |
| RBC lysis buffer(10X) | BioLegend | Cat# 420301 |
| MicroAmp Optical 96-well reaction plate | Applied biosystems | Cat# N8010560 |
| PhenoPlate-384 ULA-Coated | PerkinElmer | Cat# 1780-22141 |

| | | |
|---|---|---|
| low-attachment surface 96-well microplate | Corning | Cat# 3474 |
| low-attachment surface 6-well microplate | Corning | Cat# 3471 |
| 4-Well glass Millicell EZ Slide | Millipore | Cat# PEZGS0416 |
| 8-Well glass Millicell EZ Slide | Millipore | Cat# PEZGS0816 |
| Cell strainers (40 µm) | Fisher Scientific | Cat# 07201430 |
| Cell strainers (70 µm) | Fisher Scientific | Cat# 07201431 |
| Cell strainers (100 µm) | Fisher Scientific | Cat# 07201432 |
| Cell strainers (150 µm) | pluriStrainer | Cat# 43-50150-50 |
| Cell strainers (200 µm) | pluriStrainer | Cat# 43-50200-03 |
| Cell strainers (300 µm) | pluriStrainer | Cat# 43-50300-03 |
| Cell strainers (500 µm) | pluriStrainer | Cat# 43-50500-03 |
| **Experimental models: Cell lines** | | |
| Mouse KPC cell lines | Dr. Ronald A. DePinho's Lab | N/A |
| Human Panc-1 | ATCC | Cat# CRL-1469 |
| **Experimental models: Organisms/strains** | | |
| Mouse: C57BL/6 | Jackson Laboratories | Stock#: 000664 |
| Human pancreatic cancer samples (PDOs) | Tissue Procurement and Distribution Core of Indiana University Simon Cancer Center | Table S3 |
| **Software and Algorithms** | | |
| GraphPad | GraphPad Software Inc. | GraphPad Prism 9.0 software |
| Cytobank | Cytobank Inc. | https://premium.cytobank.org/ |
| FlowJo | FlowJo LLC | FlowJo version 10.8.0 |
| Matlab | MathWorks Inc. | Version 8.2.1, R2016b |
| IMARIS | Oxford instruments | IMARIS x64 8.1.2 |
| ImageJ | N/A | ImageJ (64bit 1.50e) |
| Aperio ImageScope | Leica Biosystems | V12.4.3.5008 |
| R software | R Software Inc. | https://www.R-project.org/ |

**Table S6. Quantitative reverse transcription PCR Primers**

| Mouse primers | | | |
|---|---|---|---|
| **Name** | **Sequence 5' to 3'** | **Name** | **Sequence 5' to 3'** |
| B2m qF | TTCTGGTGCTTGTCTCACTGA | Tapbp qF | GGCCTGTCTAAGAAACCTGCC |
| B2m qR | CAGTATGTTCGGCTTCCCATTC | Tapbp qR | CCACCTTGAAGTATAGCTTTGGG |
| Calr qF | TGGCTGCTCCCAATAATGTCT | Pdia3 qF | CGCCTCCGATGTGTTGGAA |
| Calr qR | GAGGGTAGTGACCAAAAGATGG | Pdia3 qR | CAGTGCAATCCACCTTTGCTAA |
| Erap1 qF | TAATGGAGACTCATTCCCTTGGA | Canx qF | ATGGAAGGGAAGTGGTTACTGT |
| Erap1 qR | AAAGTCAGAGTGCTGAGGTTTG | Canx qR | GCTTTGTAGGTGACCTTTGGAG |
| Sec23a qF | AGATGGGGTCCGGTTCAGTT | Hspa5 qF | ACTTGGGGACCACCTATTCCT |
| Sec23a qR | GGTAGGTCGGGTCTCTCCTT | Hspa5 qR | ATCGCCAATCAGACGCTCC |
| Sec24a qF | TCCTGTCCACAATACACTGATGT | H2-T22 qF | TCCCTTTGGGTTCACACTCG |
| Sec24a qR | GAACCACCGTAGTTCGACTGT | H2-T22 qR | AGTCGTCCATGCTCTTGTTGT |
| Sec24c qF | CTGGCCGGAATGCAGATCAG | H2-T23 qF | ACAGTCCCGACCCAGAGTAG |
| Sec24c qR | TGAGGATAGGAGCCGTATGGA | H2-T23 qR | CCACGTAGCCGACAATGATGA |
| Sec24d qF | GGAGAGGTCTTTGTTCCTTTGTT | Bcap31 qF | GCCACCTTCCTCTACGCAG |
| Sec24d qR | GTCTCTGTTCTTGAGCTTCCC | Bcap31 qR | TGCCATAGGTCACTACCAACTC |
| Sec31a qF | CAGTCCTCACTTACGCTAAACC | Sec13 qF | GAACACTGTGGACACCTCTCA |
| Sec31a qR | ATTCCCTGCACAGATGTAGCA | Sec13 qR | CTCCATTCCGCACATCGAAAA |
